# Inter-individual methylation variability in differentially methylated regions between maternal whole blood and first trimester CVS

**DOI:** 10.1186/s13039-014-0073-8

**Published:** 2014-11-01

**Authors:** Marios Ioannides, Elisavet A Papageorgiou, Anna Keravnou, Evdokia Tsaliki, Christiana Spyrou, Michael Hadjidaniel, Carolina Sismani, George Koumbaris, Philippos C Patsalis

**Affiliations:** The Cyprus Institute of Neurology and Genetics, Nicosia, Cyprus; Department of Biological Sciences, University of Cyprus, Nicosia, Cyprus; NIPD Genetics Ltd, Nicosia, Cyprus

**Keywords:** Non-invasive prenatal diagnosis, Inter-individual variability, Differentially methylated regions, MeDIP

## Abstract

**Background:**

DNA methylation is the most studied form of epigenetic regulation, a process by which chromatin composition and transcription factor binding is altered to influence tissue specific gene expression and differentiation. Such tissue specific methylation patterns are investigated as biomarkers for cancer and cell-free fetal DNA using various methodologies.

**Results:**

We have utilized methylation DNA immunoprecipitation (MeDIP) and real-time quantitative PCR to investigate the inter-individual methylation variability of differentially methylated regions (DMRs) on chromosomes 18 and 21. We have characterized 15 newly selected and seven previously validated DMRs in 50, 1^st^ trimester Chorionic villus samplings (CVS) and 50 female non-pregnant peripheral blood (WBF) samples. qPCR results from MeDIP and genomic DNA (Input) assays were used to calculate fold enrichment values for each DMR. For all regions tested, enrichment was higher in CVS than in WBF samples with mean enrichments ranging from 0.22 to 6.4 and 0.017 to 1 respectively. Despite inter-individual variability, mean enrichment values for CVS were significantly different than those for WBF in all DMRs tested (p < 0.01). This observation is reinforced by the absence of overlap in CVS and WBF enrichment value distributions for 15 of 22 DMRs.

**Conclusions:**

Our work provides an expansion in the biomarker panel available for non-invasive prenatal diagnosis (NIPD) using the MeDIP-qPCR methology for Down syndrome and can eventually provide the starting point towards the development for assays towards the detection of Edwards syndrome. Furthermore, our data indicate that inter-experimental and inter-individual variation in methylation is apparent, yet the difference in methylation status across tissues is large enough to allow for robust tissue specific methylation identification.

**Electronic supplementary material:**

The online version of this article (doi:10.1186/s13039-014-0073-8) contains supplementary material, which is available to authorized users.

## Background

In vertebrates DNA methylation is a conserved epigenetic modification by which DNA methyltransferases add a methyl group to carbon 5 of cytosine residues present in CpG dinucleotides. This modification is the most studied form of epigenetic regulation and has been strongly associated with chromosomal stability and imprinting control [1]. Furthermore, this epigenetic process also regulates chromatin composition and transcription factor binding to directly influence transcriptional activity [2,3].

DNA methylation occurs primarily in CpG islands (GGIs) and shores both in coding and non-coding regions of the genome, with gene regulatory regions such as promoters and first exons being a frequent methylation target [4]. Due to this integral relationship with gene expression regulation, DNA methylation patterns are very closely associated with developmental processes and differentiation. Consequently, DNA methylation directly modulates phenotype, and distinct methylation patterns have been associated with tissue specificity and a variety of disease states ranging from cancer to neurological disorders [5,6]. These tissue specific differentially methylated regions (tDMRs) are currently under investigation for their utility as biomarkers for disease progression and prognosis, particularly in the field of cancer research, disease detection and response to treatment [7].

The discovery of cell free fetal DNA (cffDNA) in the maternal circulation has greatly facilitated the development of non-invasive prenatal diagnosis (NIPD) [8]. The direct correlation between phenotype and DNA methylation patterns has allowed the use of DMRs as possible biomarkers in prenatal diagnosis. Several groups have utilized the methylation differences between placenta-derived cffDNA and maternal DNA in order to identify highly specific fetal DMR biomarkers for non-invasive prenatal diagnosis of aneuploidies. Previous studies employed a variety of methods including sodium bisulfite conversion and methylation sensitive restriction digestion, but yielded a relatively small number of fetal specific DMRs including the *SERPINB5*, *RASSF1A* and U-*PDE9A* genes [9-11].

In 2009, Papageorgiou et al. [12] applied methylation DNA immunoprecipitation (MeDIP) coupled with high resolution tiling oligonucleotide array (Chip) analysis to identify DMRs between Chorionic villus sampling (CVS) and female peripheral blood DNA (WBF). They were able to identify thousands of DMRs on chromosomes 13, 18, 21, X, and Y including methylation sensitive restriction sites, CGIs and promoter regions. This MeDIP-Chip approach was the trigger for investigating the utility of MeDIP followed by real-time qPCR (MeDIP-qPCR) for the non-invasive prenatal diagnosis of trisomy 21, yielding 100% sensitivity and specificity [13]. This novel NIPD method was validated by a second study of 175 cases again yielding high sensitivity and specificity [14].

The current study utilizes the MeDIP-qPCR methodology to expand our range of fetal specific DMR biomarkers by selecting and screening 15 additional DMRs on chromosomes 21 and 18. Special emphasis is given on investigating the methylation variability in different samples from these newly selected and previously reported DMRs [12-14] by screening them in a set of 50, 1^st^ trimester CVS and 50 WBF. Overall, this work confirms the distinctively different methylation status of these regions in CVS and WBF.

## Results

Using the above criteria we identified a set of 40 candidate DMRs between CVS and WBF from the microarray data [12]. This set was subsequently screened in a cohort of six CVS and six WBF to calculate the enrichment values for each DMR (Additional file 1). Based on this initial screening we were able to select the 15 regions with the highest CVS enrichment for further validation/characterization, using seven previously validated DMRs by Papageorgiou et al. [13] and Tsaliki et al. [14], as a comparison standard.

This DMR validation study was conducted on a set of 50 CVS and 50 WBF samples using the MeDIP–qPCR methodology (Table 1), the efficiency of which was monitored using one hypermethylated (HYPER) and one hypomethylated (HYPO) control regions. The HYPER is a region that showed hypermethylation for both CVS and WBF, while the HYPO is a region that showed hypomethylation for the two tissues [12]. Enrichment values for HYPO were low in WBF and CVS samples while the HYPER control region showed enrichment for CVS and WBF with mean enrichment values of 3.12 and 3.22 respectively, indicating that the MeDIP procedure was highly specific for the methylated regions. Moreover, the previously validated DMRs performed as previously described [12], exhibiting distinctively different enrichment between CVS and WBF.

**Table 1:** **Ranking of DMRs tested according to the difference between mean enrichment values for each DMRs**

**Marker**	**Mean WBF**	**Mean CVS**	**Mean difference**	**SD WBF**	**SD CVS**	**U pval**	**Coefficient of variation WBF**	**Coefficient of variation CVS**
**EI-4**	0.017	6.384	6.367	0.022	2.143	1.53E-17	1.294	0.336
**EII-1**	0.065	5.319	5.254	0.071	1.937	7.06E-18	1.092	0.364
**H2**	0.135	4.068	3.933	0.093	1.252	7.06E-18	0.689	0.308
**EI-2**	0.064	3.894	3.83	0.2	1.338	7.50E-18	3.125	0.344
**EI-3**	0.116	3.905	3.789	0.312	1.556	1.96E-17	2.690	0.398
**B3**	0.126	3.86	3.734	0.1	1.268	2.29E-17	0.794	0.328
**M27**	0.532	4.113	3.581	0.2	1.386	7.06E-18	0.376	0.337
**D2**	0.317	3.364	3.047	0.179	1.301	7.06E-18	0.565	0.387
**M28**	0.189	2.777	2.588	0.125	0.919	7.06E-18	0.661	0.331
**M1E**	0.149	2.636	2.487	0.097	0.742	3.44E-17	0.651	0.281
**Id1**	0.398	2.682	2.284	0.155	0.932	1.04E-17	0.389	0.348
**A5**	0.337	2.505	2.168	0.159	0.986	7.06E-18	0.472	0.394
**C5**	0.18	2.321	2.141	0.106	0.84	7.06E-18	0.589	0.362
**C1**	0.106	2.229	2.123	0.083	0.635	7.06E-18	0.783	0.285
**AII-2**	0.065	2.003	1.938	0.084	0.933	1.23E-16	1.292	0.466
**On2**	0.281	1.993	1.712	0.138	0.552	7.06E-18	0.491	0.277
**Nn2**	0.245	1.924	1.679	0.107	0.78	7.07E-18	0.437	0.405
**J2**	0.116	1.707	1.591	0.079	0.519	7.06E-18	0.681	0.304
**Fd1**	0.135	1.676	1.541	0.101	0.513	7.06E-18	0.748	0.306
**M25**	1.038	1.822	0.784	0.452	0.655	1.88E-09	0.435	0.359
**M20**	0.42	0.796	0.376	0.186	0.303	2.37E-10	0.443	0.381
**M18**	0.097	0.22	0.123	0.07	0.151	6.36E-08	0.722	0.686
**HYPER**	3.124	3.226	0.102	0.68	0.982	0.951	0.218	0.304
**HYPO**	0.469	0.508	0.039	0.945	0.948	0.119	2.015	1.866

Despite the statistical significance of all enrichment values (p < 0.01), the four markers (M25, M20, M18, Fd1) that showed the lowest difference were not selected as potential DMRs.

**Figure 1:**
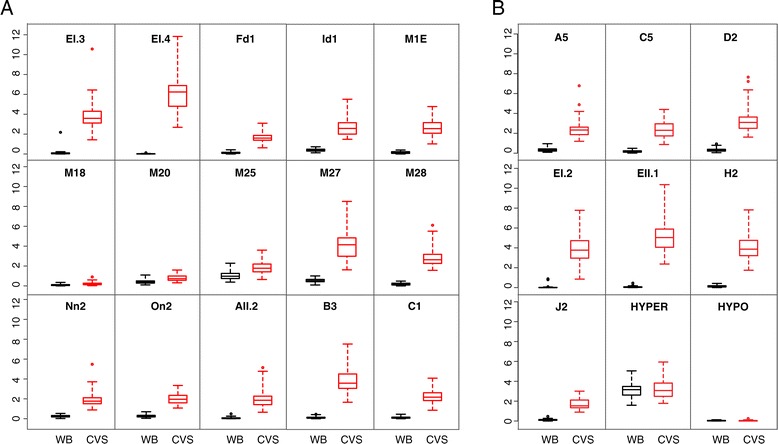
**Enrichment profile for all DMRs on 50 WBF and 50 first trimester CVS.** Box plots show the distribution of the relative fold enrichment values for WBF (black) and CVS (red) for each DMR. The median value is represented by a horizontal line. The bottom of the box indicates the 25^th^ percentile (lower quartile) and the top the 75^th^ percentile (upper quartile). Whisker lines indicate the outlier boundaries [top: median + 3(75%-25%); bottom: median -3(75%-25%)]. **A**: 11 of 15 newly characterized DMRs show clear separation of the methylation enrichment values between WBF and CVS despite the methylation variability. Fd1, M18, M20, M25 show overlap between the interquartile values, thus they were not characterized as DMRs. **B**: Previously validated DMRs used as a comparison standard show clear separation between WBF and CVS. HYPER: hypermethylated marker for both tissues, HYPO: Hypomethylated marker for both tissues.

All tested DMRs showed a significant enrichment (p < 0.01) in CVS compared to those of WBF (Table 1). We compared the performance of the 15 newly selected DMRs with the previously validated set and we were able to determine that 11 of 15 DMRs showed enrichment values higher than the lowest of the previously validated DMRs, ranging from 1.9 to 6.4. Additional comparison of the two DMR sets also illustrated that for 11 of these 15 regions the difference of means (mean enrichment CVS – mean enrichment WBF) was again higher than the respective values of the validated DMRs (ranging from 1.6 to 6.4) (Table 1). Our analysis also shows that the enrichment distributions for CVS and WBF have no overlap for these 11 DMRs (Figure 1).

**Figure 2:**
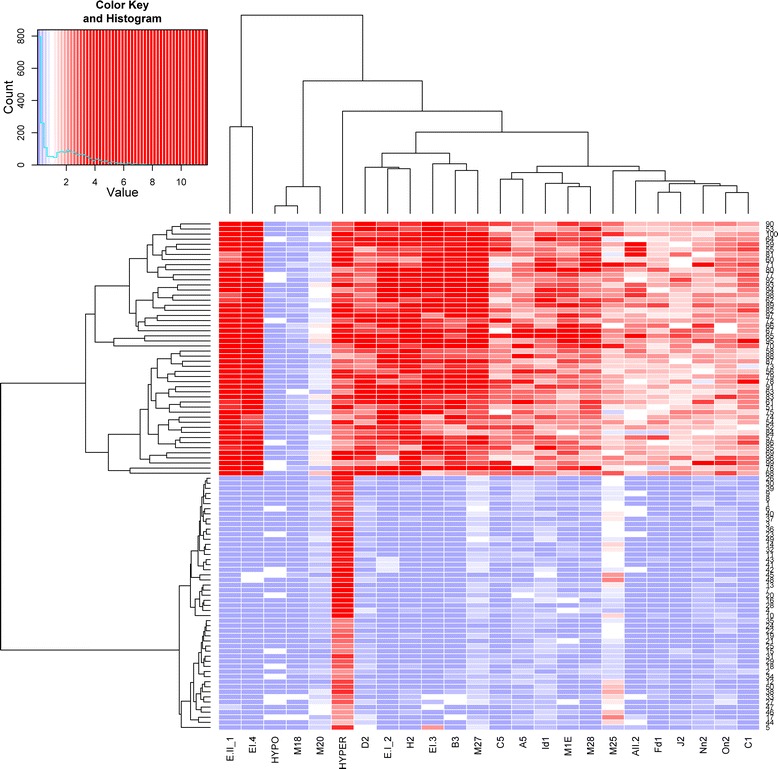
**DMRs exhibiting tissue specificity between CVS and WBF.** A heat map constructed based on the relative fold enrichment values obtained for the two tissues tested (CVS and WBF) with the MeDIP-qPCR approach shows clustering of the hypermethylated CVS samples (high enrichment-red) and the hypomethylated WBF samples (low enrichment–blue). Furthermore, DMR clustering analysis showed no cluster distinction between the 15 newly selected DMRs and the previously validated DMRs that were used in this study as a comparison standard.

To better investigate tissue specificity (CVS-WBF) in the 15 newly selected DMRs in relation to the previously validated DMRs, we also constructed a heat map and hierarchical clustering of the 50 CVS and 50 WBF samples based on the obtained enrichment values (Figure 2). This analysis shows a clear differentiation between the two tissue types based on the obtained enrichment values. Furthermore, DMR clustering analysis showed that there was no distinct clustering separation between the newly selected and the previously validated DMRs.

## Discussion

Our study aimed to validate and characterize a set of differentially methylated regions between CVS and WBF, obtained from MeDIP-Chip data [12]. The methylation characteristics of the 15 candidate DMRs, located on chromosomes 18 and 21, were ascertained in 50 CVS and 50 WBF using the MeDIP-qPCR methodology. To our knowledge this is the first MeDIP based biomarker screening study utilizing such a large sample set. None of the selected DMRs were located on CGIs, but within intergenic or intragenic regions. Such DMR distribution in non-coding intergenic and intragenic sequences is in agreement with data from a large scale investigation of tissue specific methylation profiles by Rakyan et al. [15], who reported differential methylation of intergenic and CpG poor promoter regions in addition to CGIs.

Based on our results, all 15 DMRs showed differential enrichment between the two tissues and 11 out of these 15 were strongly and consistently hypermethylated in CVS samples. The ability of these DMRs to distinguish between CVS and WBF was equivalent to that of the seven previously validated DMRs used as performance standards. In fact, the tissue discriminating performance of the DMRs tested here, shows close similarities with the previously validated DMRs as it is illustrated by our heat map distribution and the unsupervised clustering patterns obtained.

DMR enrichment values showed variability among the different samples. This is likely caused by combination of both inter-experimental technical variability and inter-individual methylation variability. The presence of variability in MeDIP based assays has previously been described by Butcher et al. [16]. In addition, the issue of inter-individual DNA methylation variability has been the focus of several studies [17-19]. This high inter-individual variability has been attributed to a variety of factors including environmental conditions, diet, age and psychosocial factors [20-23]. Furthermore, it has been documented that regions with low CpG density, as the DMRs under investigation, show higher inter-individual variability as compared to regions with high CpG density, such as CGIs [24].

Others have also shown that methylation variability can coincide with tissue specific DMRs without obscuring the tissue discriminating properties of those DMRs [17]. It is therefore of no surprise that despite the DNA methylation variability in our study, the newly validated set of 11 DMRs clearly distinguishes between CVS and WBF tissues.

Our work here substantially increases the number of confirmed chromosome 21 fetal specific DMRs, and therefore provides a significant expansion in the biomarker panel available for MeDIP-qPCR-based NIPD of Down syndrome. Such an expansion is predicted to further improve the robustness of the methodology and bolster its diagnostic classification power. It is also very important to note that our current study is the first to validate chromosome 18 fetal specific DMRs in a relatively large sample set. This small panel of chromosome 18 DMRs can potentially provide a very valuable testing platform on which future NIPD assays for Edwards syndrome will be developed.

## Conclusions

NIPD has gained a lot of interest the last few years. Utilizing the methylation differences between fetal and maternal DNA, several groups have managed to identify biomarkers using different approaches. This study aimed to characterize and validate fetal specific methylated regions using the MeDIP-qPCR methodology. We were able to show that the selected regions had distinct methylation patterns between fetal and maternal tissue, despite inter-individual and inter-experimental variability. In addition, we have expanded the panel of the existing DMRs on chromosome 21 and have characterized a new set of markers on chromosome 18 which can provide the starting point towards the development for assays towards the detection of Edwards syndrome.

## Methods

### Human Samples and DNA preparation

WBF samples were obtained anonymously from 50 normal non-pregnant females 20-40 years of age. Fifty, 1^st^ trimester CVS were obtained from the Department of Cytogenetic and Genomics at the Cyprus Institute of Neurology and Genetics (Nicosia, Cyprus). Protocols used for collecting samples for our study were approved by the appropriate Bioethics Committees, and informed consent was obtained from all participants. WBF and CVS samples were used to extract DNA using the QIAamp DNA blood midi kit or the QIAmp DNA mini kit according to the manufacturer’s instructions (QIAGEN, Hilden, Germany). All CVS underwent karyotyping and Quantitative-Fluorescent PCR (QF-PCR) analysis in order to confirm their normal status.

### Ligation-mediated PCR (LM-PCR) and MeDIP assay

LM-PCR and MeDIP assays were conducted as described previously [12]. Briefly, 2.5 μg of genomic DNA were sonicated using the Bioruptor Twin sonication system (UCD-400, Diagenode, Liege, Belgium) into fragments, 300-1000 bp in size. Fragment size was verified using agarose gel electrophoresis. The fragments were blunt-ended using HPLC water, 1X NEB buffer 2 (New England BioLabs, Ipswich, UK), 10X bovine serum albumin (New England BioLabs) 100 mmol/L dNTP mix (GE Healthcare, Little Chalfont, UK) and T4 DNA polymerase (3 U/μl; New England BioLabs). Fragments were purified using the QiAquick PCR purification kit (Qiagen) and linkers were then ligated onto the blunt ends by overnight incubation at 16°C with T4 DNA ligase (New England) and T4 DNA ligase buffer (New England). Overhangs were subsequently filled in by incubating at 72°C for 10 minutes with 100 mmol/L dNTP mix (GE Healthcare), 1X PCR gold buffer (Roche, Mannheim, Germany), 1.5 mmol/L MgCl_2_ (Roche) HPLC water and AmpliTaq DNA polymerase (Applied Biosystems, Branchburg, New Jersey, USA). 50 ng of ligated DNA was removed and kept as input DNA. The remaining ligated DNA (800-1200 ng) was subjected to MeDIP using 3 μg mouse anti-5’methylCytosine (a-5mC) antibody (Eurogentec Saraing, Belgium). Hypermethylated DNA bound to a-mC antibodies was magnetically captured using Dynabeads® M-280 Sheep Anti-Mouse IgG magnetic beads (Life technologies, Carlsbad, California, USA) and subsequently released using proteinase K (Roche). LM-PCR used 12 ng of each input and MeDIP DNA as described earlier [12].

### DMR selection

Candidate DMRs on chromosomes 18 and 21 were selected from a set of potential differentially methylated regions previously described [12] according to the following three criteria: a) the region included at least three consecutive microarray probes, b) array results showed consistent DNA hypermethylation in first and third trimester placentas and hypomethylation in WBF samples, c) the region did not include segmental duplications and copy number variable regions based on the Database for Genomic Variants (DGV) [25]. The regions considered for this paper are shown in Table 2.

**Table 2:** **Characteristics of the regions tested**

**Chromosomal region**	**Position (hg18)**	**Location type**	**Gene involved**
Nn	chr21:31426757-31427146	Intragenic	TIAM1
**H**	**chr21:32268787-32269137**	**Intragenic**	**HUNK**
**C**	**chr21:33320530-33320815**	**CpG Island**	**OLIG2**
On	chr21:34492714-34493203	Intergenic	
**J**	**chr21:37841231-37841506**	**Intergenic**	
**A**	**chr21:39279691-39279971**	**Intergenic**	
Fd	chr21:42005961-42006216	Intragenic	C21orf129
M27	chr21:42178808-42179008	Intragenic	C2CD2
**D**	**chr21:42189235-42189849**	**LINE-L1**	**C2CD2**
**EI**	**chr21:42355366-42355908**	**Intergenic**	
**EII**	**chr21:42357141-42357401**	**Intergenic**	
Id	chr21:42753677-42754026	Intergenic	
M1E	chr21:44953640-44953854	Intragenic	TSPEAR
M28	chr21:45171015-45171225	Intragenic	ITGB2
AI	chr18:55086179-55086755	Intragenic	RAX NM-013435
AII	chr18:55090284-55090605	Intragenic	RAX NM-013435
B	chr18:44165984-44166275	Intergenic	
C	chr18:58955844-58956604	Intragenic	BCL2NM-000633
M18	chr21:15331818-15331945	Intragenic	NR1P1
M20	chr21:15178413-15178497	Intergenic	
M25	chr21:37692864-37692974	Intergenic	DYRK1A

Regions in bold indicate previously validated regions [13,14].

### Real-Time quantitative PCR (qPCR)

Primer design, optimal primer concentration experiments, and efficiency (e) of each qPCR reaction were performed as previously described [12] with the following modifications. Each qPCR reaction was performed on 8 ng of template DNA using SYBR Green PCR mastermix (Eurogentec) in a final reaction volume of 10 μl, using a BIORAD CFX 384 Real time system (BIORAD, Hercules, California). Each MeDIP, or Input template DNA was used to prepare three replicate qPCR reactions that were used for calculating the average Ct value for each template. Primer3 software [26] was used to design the qPCR primer sets that were synthesized by Sigma-Aldrich (Munich, Germany). Primer sets utilized for this body of work are listed in Table 3.

**Table 3:** **Primer sequences on DMRs tested**

**Primer**	**Forward**	**Reverse**	**Position**
CHR21(M27)	ATACGTGTCCTGCCTTCCAC	GCTTTGAGCAGAGAGGGAAA	42178812-42178948
CHR21(M28)	CCCAGAAATTCCATTTGCAG	GAAAGGCTCAACCAACCAAC	45171107-45171192
CHR21(M1E)	TCGCACTGAGGCTTCCTACT	AAGTTGTGGGCTGGGATTTT	44953674-44953772
CHR21(Nn2)	ACCATTGTGGATCACAGCAG	GCTCCGAGGATTAGGGAAAG	31427008-31427139
CHR21(On2)	CTCCTGACCCACTCCCAATA	GGAAACTCAGGGTCAAACGA	34492982-34493090
CHR21(Fd1)	ATGTTGCCTGGGATATGCTT	AACTGGCTGCGTGAGGATA	42006045-42006153
CHR21(EI-3)	GCCTTGGGACAAAAATGACA	TGGGCACAGCCCTAACTAAC	42355352-42355484
CHR21(EI-4)	GGCCAGGTTGTTTCAGATTG	TTCCGGCAGAGTTTATTTGG	42355802-42355908
CHR21(Id1)	ACCGTATCATTTCCCCAGGT	TGACCACATTTCCACCACAG	42753720-42753866
**CHR21(A5)**	**GCTGGACCAGAAAGTGTTGAG**	**GTGTGCTGCTTTGCAATGTG**	**39279856-39280004**
**CHR21(C5)**	**CTGTTGCATGAGAGCAGAGG**	**CGTCCCCCTCGCTACTATCT**	**33320735-33320829**
**CHR21(D2)**	**TGCAGGATATTTGGCAAGGT**	**CTGTGCCGGTAGAAATGGTT**	**42189557-42189683**
**CHR21(EI-2)**	**TGAATCAGTTCACCGACAGC**	**GAAACAACCTGGCCATTCTC**	**42355712-42355815**
**CHR21(EII-1)**	**CCGTTATATGGATGCCTTGG**	**AAACTGTTGGGCTGAACTGC**	**42357215-42357341**
**CHR21(H2)**	**CCACATCCTGGCCATCTACT**	**TTCCACAGACAGCAGAGACG**	**32268843-32268943**
CHR21(M18)	GATGGATGGCCTTTTGGTAA	TATTTGGTTTGCCCCTTCCT	15331818-5331945
CHR21(M20)	CATTAGCGGGTCAGCTAGGA	TGGCAATTACATCTGCCATTA	15178413-5178497
CHR21(M25)	TTGTCTGCCCGTATGGAAGT	ATGGTTGTAGGGCTCATTCA	37692864-37692974
**CHR21(J2)**	**ATTCTCCACAGGGCAATGAG**	**TTATGTGGCCTTTCCTCCTG**	**37841284-37841411**
CHR18(AII2)	TGTGCCTCTCCCTTGAGACT	AAATTGCAGCCAATGCTTCT	55090427-55090524
CHR18(B3)	TGTGGTTTCAAACATGCACA	CTGAAAAGGCCACTCTGAGG	44166131-44166263
CHR18(C1)	GTGAGAGAGAACGCCAGGAG	TGAGCCAACTCTGGTGTCAG	58956266-58956391
HYPER	CAGGAAAGTGAAGGGAGCTG	CAAAACCCAATGGTCAATCC	19991387-19991465
HYPO	AGGTGCCCAATTCAAGGTA	CTTCCCCACCAGTCTTGAAA	30214952-30215055

Regions in bold indicate previously validated regions [13,14].

### Statistical calculations

MeDIP enrichment values of the CVS and WBF samples were calculated for each region using the following equation:

**Enrichment = e**^**∆Ct**^ where e corresponds to the efficiency obtained in each real-time PCR reaction e = 10^(-1/slope of STD curve)^) and ∆Ct indicates the cycle difference between input DNA and MeDIP DNA [Ct_(IN)_ – Ct_(IP)_ ].

The mean enrichment values of each DMR were compared between WBF and CVS samples using the Mann-Whitney U tests [27] and the corresponding p-values were used to decide whether there was significant evidence to claim that the mean enrichments of the two groups were different.

Hierarchical clustering of the DMRs was conducted using an iterative algorithm that joins similar clusters based on the set of dissimilarities of the 100 individuals (calculating the Euclidean distanced between clusters) and re-computing their distances at each stage by the Lance-Williams dissimilarity update formula [28].

## Additional file

Additional file 1:
**Initial screening on six WBF and six CVS for the selection of new DMRs.**
