# Supplementary material for: Inter-individual methylation variability in differentially methylated regions between maternal whole blood and first trimester CVS
Source: Mol Cytogenet. 2014 Nov 1;7:73. doi: 10.1186/s13039-014-0073-8 (PMC4243368; doi:10.1186/s13039-014-0073-8)
Supplement: Additional file 1: — Initial screening on six WBF and six CVS for the selection of new DMRs. [file 13039_2014_73_MOESM1_ESM.pdf]

Initial screening on six WBF and six CVS for the selection of new DMRs

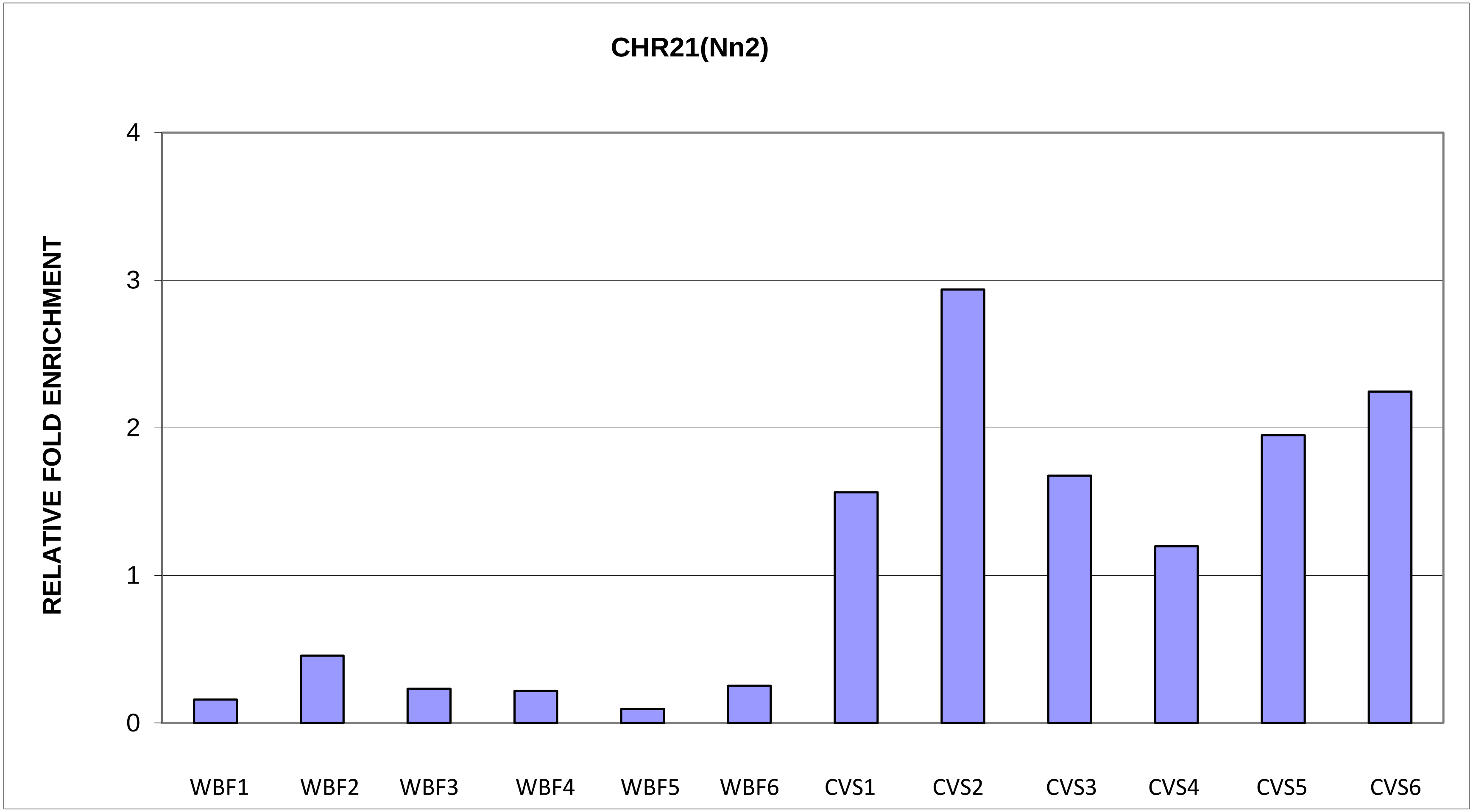
CHR21(Nn2)
RELATIVE FOLD ENRICHMENT
4
3
2
1
0
WBF1
WBF2
WBF3
WBF4
WBF5
WBF6
CVS1
CVS2
CVS3
CVS4
CVS5
CVS6

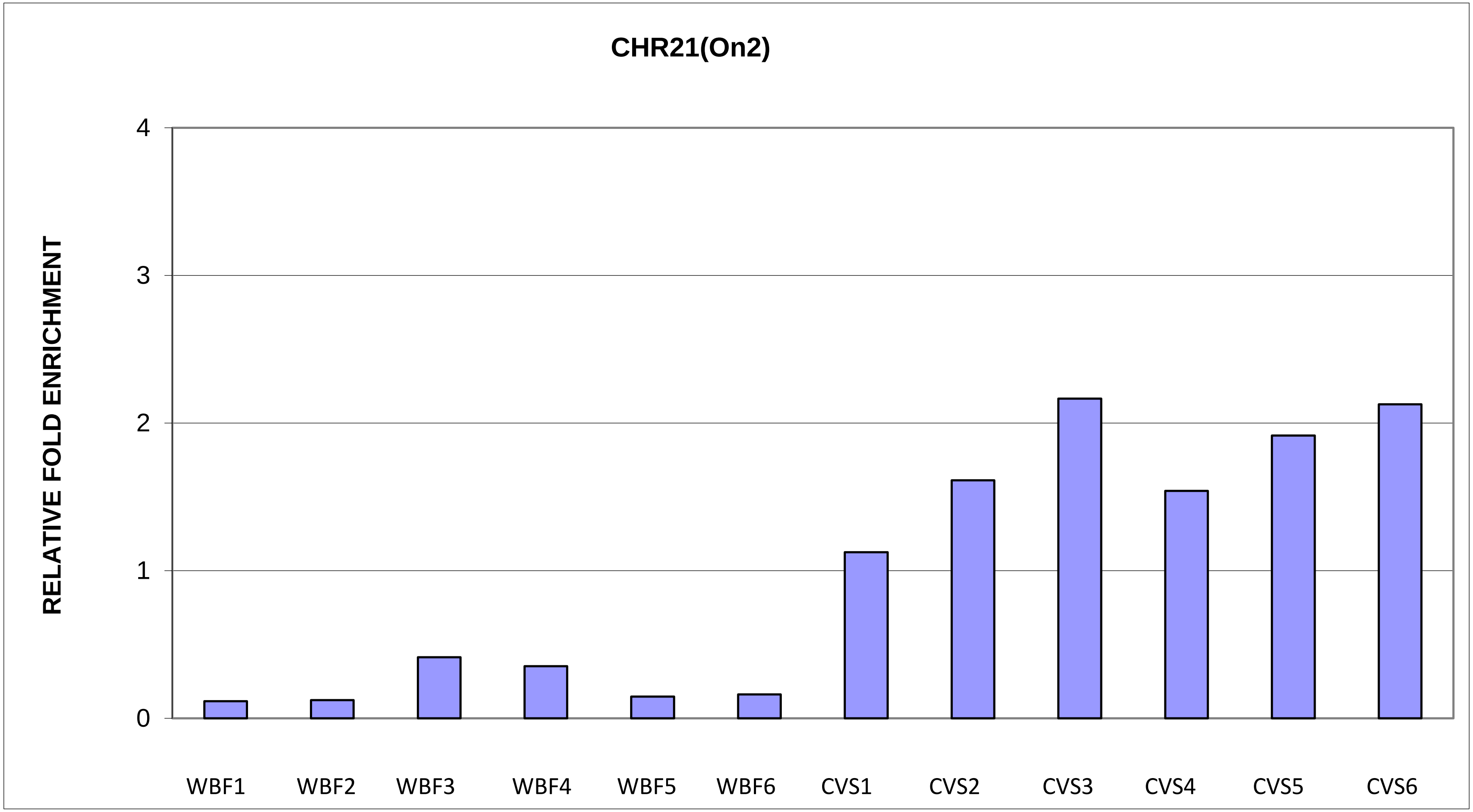
CHR21(On2)
RELATIVE FOLD ENRICHMENT
4
3
2
1
0
WBF1
WBF2
WBF3
WBF4
WBF5
WBF6
CVS1
CVS2
CVS3
CVS4
CVS5
CVS6

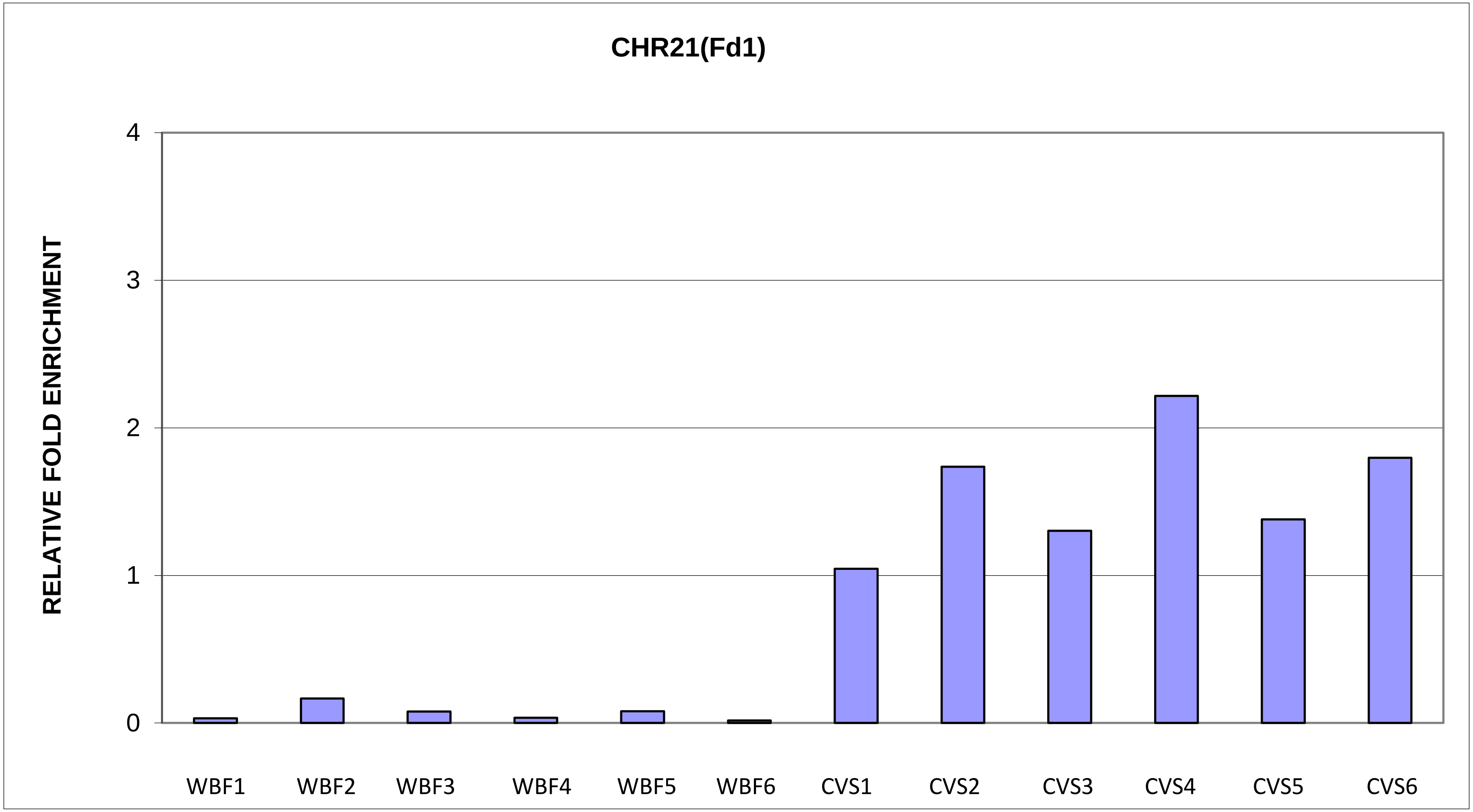
CHR21(Fd1)
RELATIVE FOLD ENRICHMENT
4
3
2
1
0
WBF1
WBF2
WBF3
WBF4
WBF5
WBF6
CVS1
CVS2
CVS3
CVS4
CVS5
CVS6

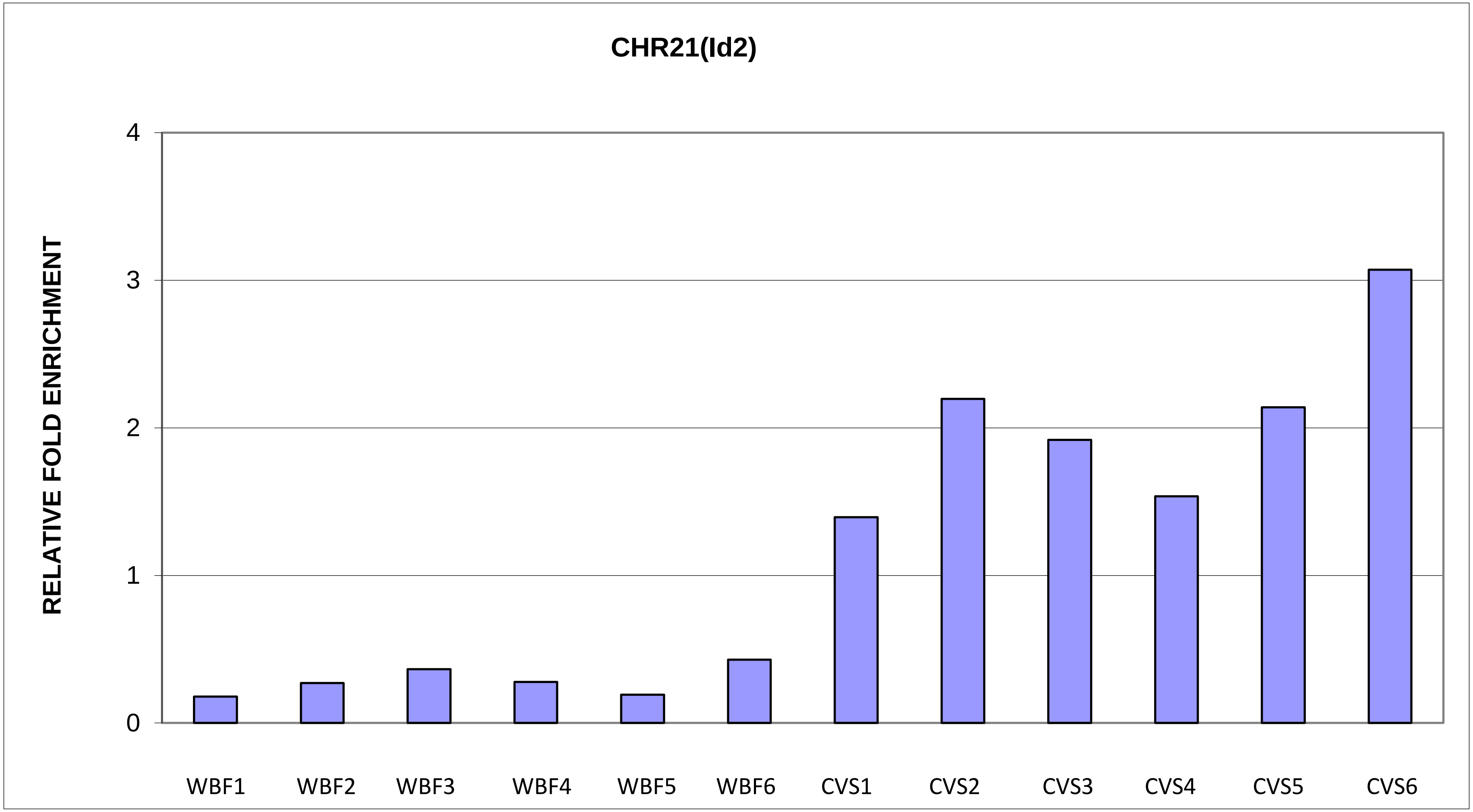

CHR21(Id2)
RELATIVE FOLD ENRICHMENT
4
3
2
1
0
WBF1
WBF2
WBF3
WBF4
WBF5
WBF6
CVS1
CVS2
CVS3
CVS4
CVS5
CVS6

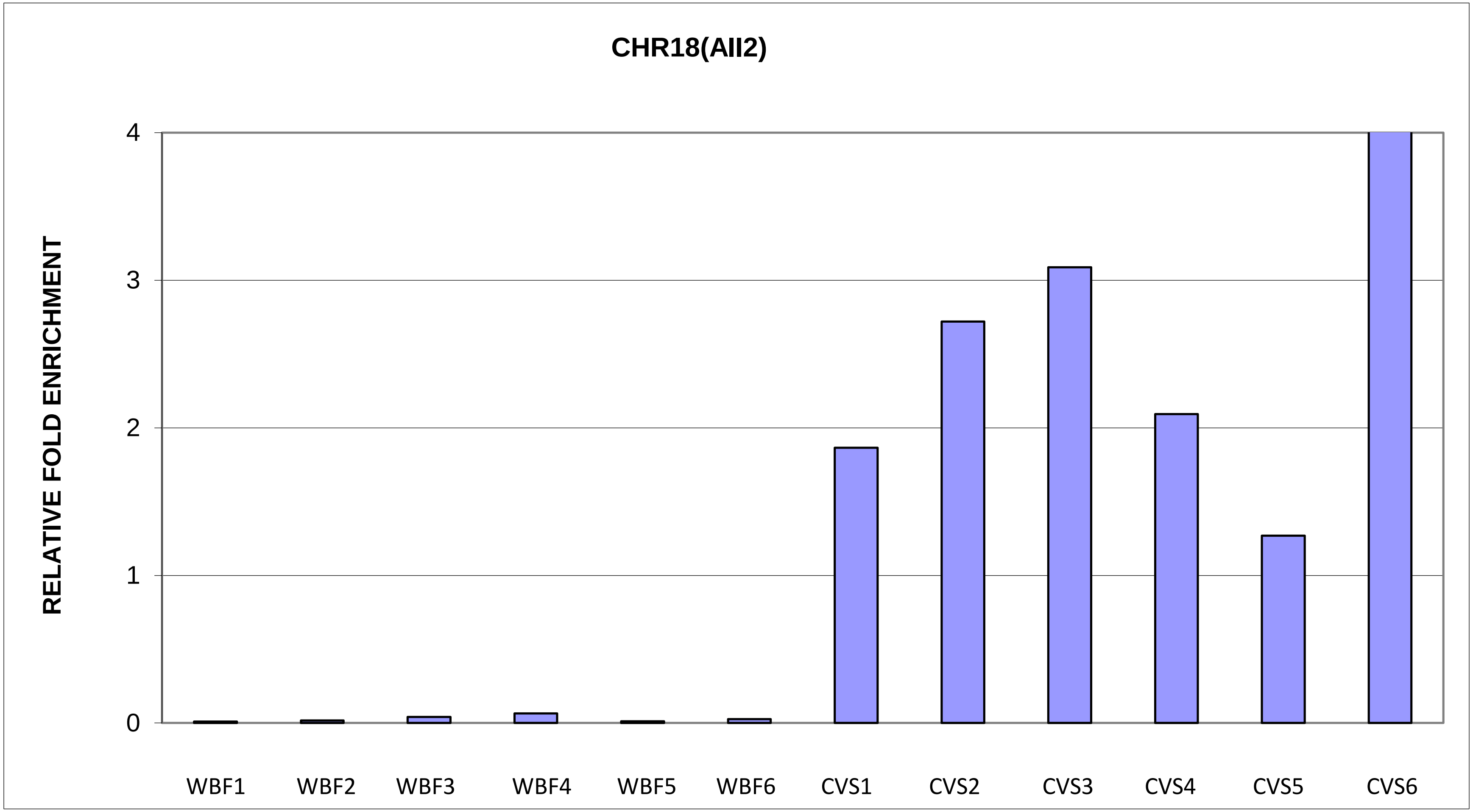
CHR18(AII2)
RELATIVE FOLD ENRICHMENT
4
3
2
1
0
WBF1
WBF2
WBF3
WBF4
WBF5
WBF6
CVS1
CVS2
CVS3
CVS4
CVS5
CVS6

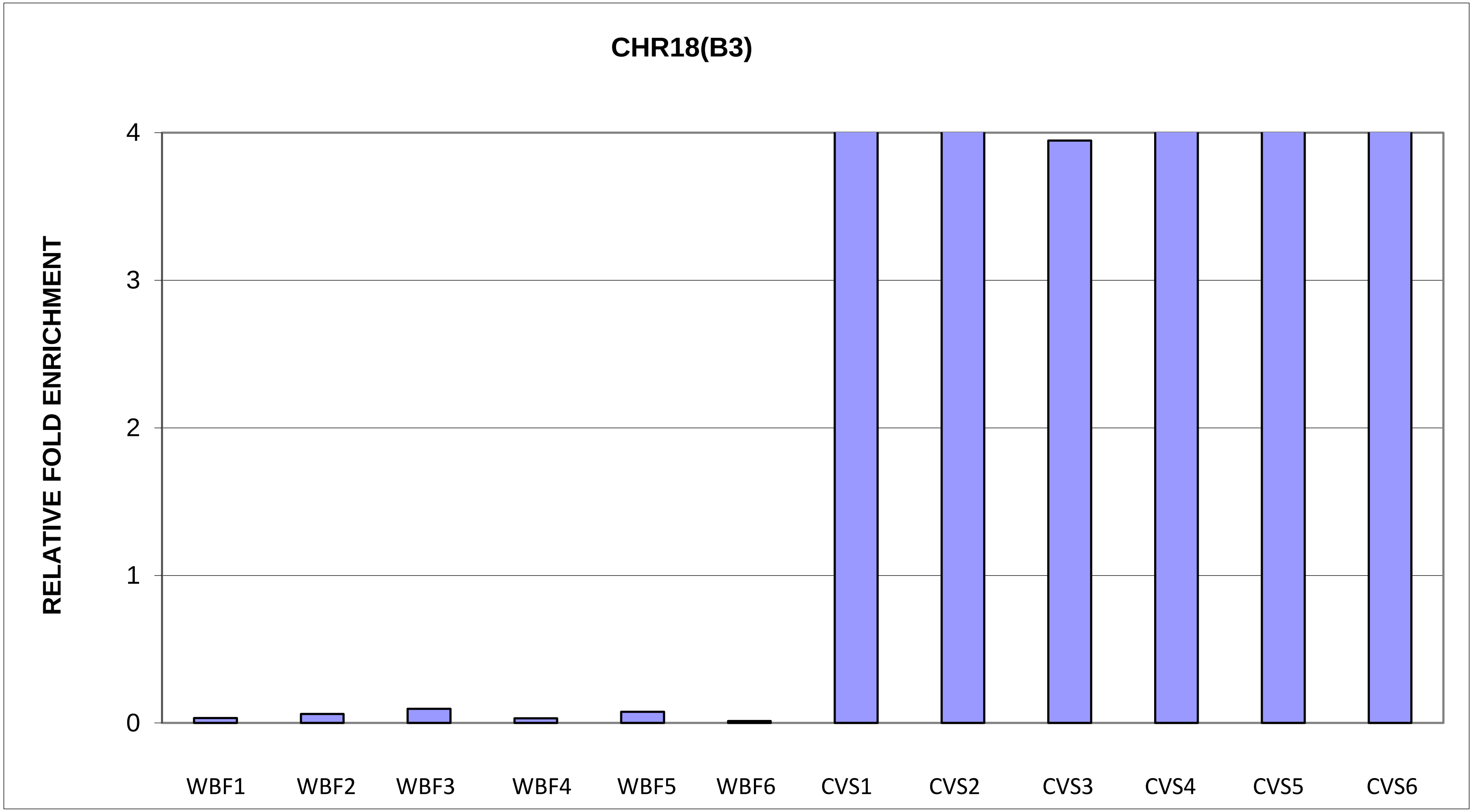
CHR18(B3)
RELATIVE FOLD ENRICHMENT
4
3
2
1
0
WBF1
WBF2
WBF3
WBF4
WBF5
WBF6
CVS1
CVS2
CVS3
CVS4
CVS5
CVS6

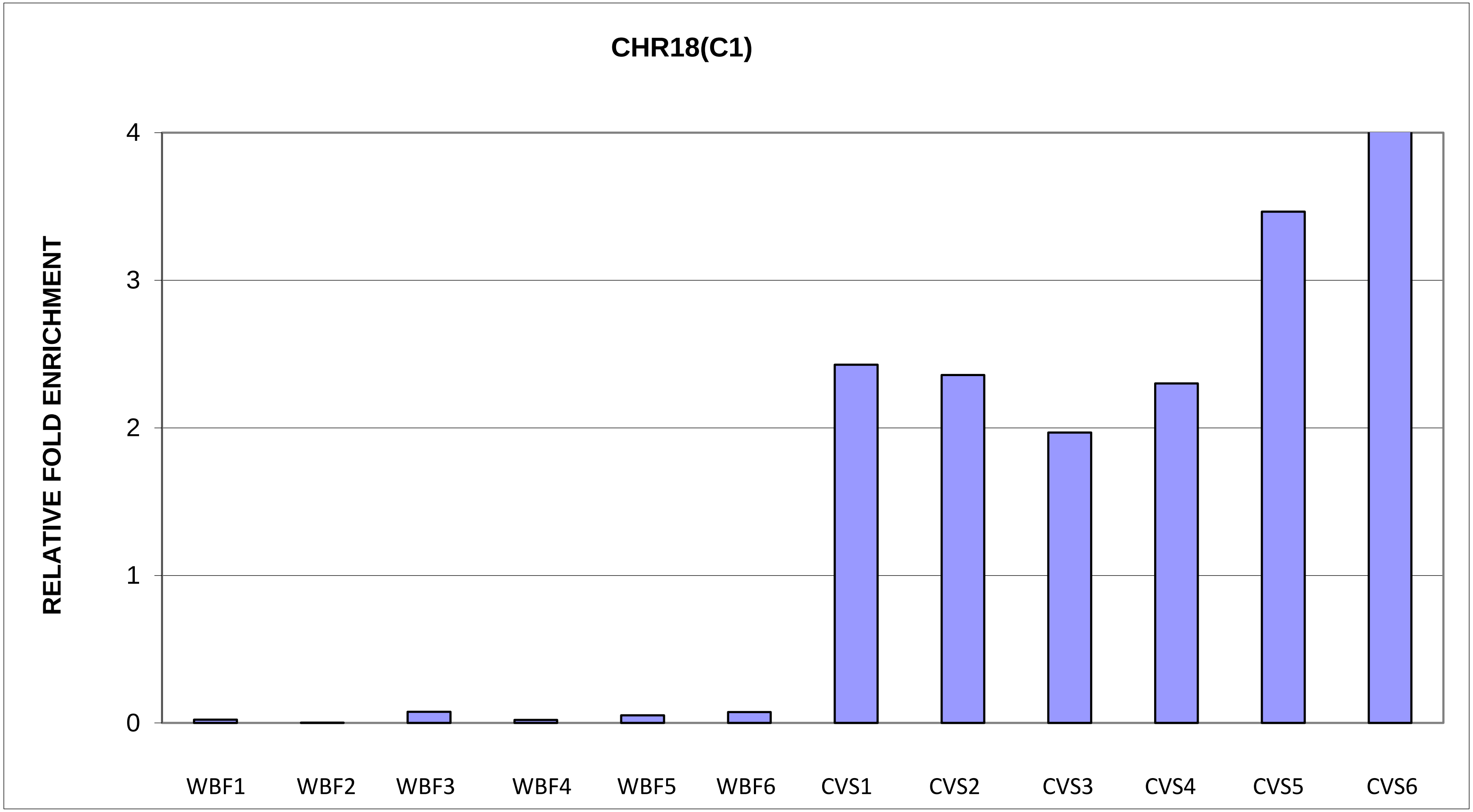
CHR18(C1)
RELATIVE FOLD ENRICHMENT
4
3
2
1
0
WBF1
WBF2
WBF3
WBF4
WBF5
WBF6
CVS1
CVS2
CVS3
CVS4
CVS5
CVS6

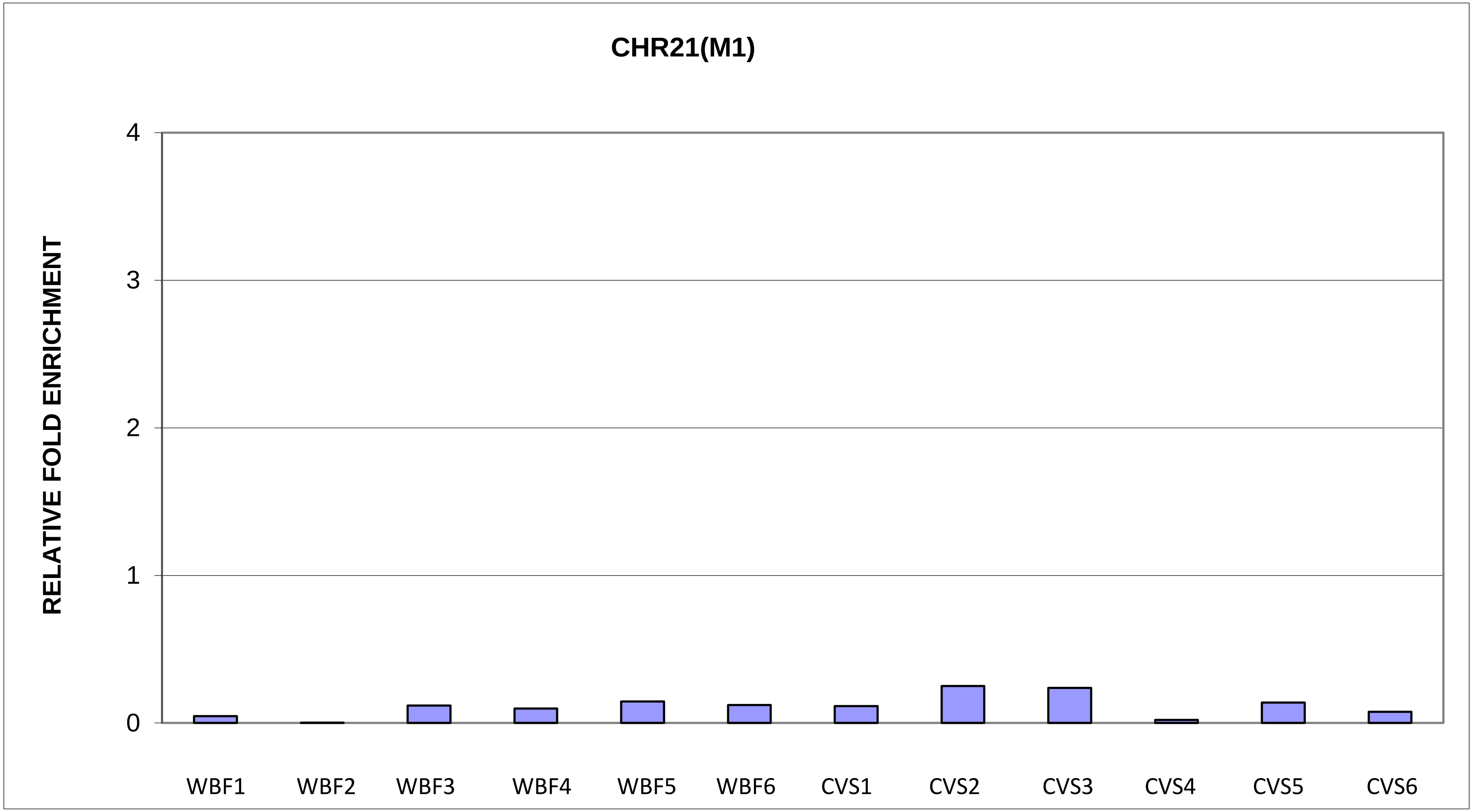
CHR21(M1)
RELATIVE FOLD ENRICHMENT
4
3
2
1
0
WBF1
WBF2
WBF3
WBF4
WBF5
WBF6
CVS1
CVS2
CVS3
CVS4
CVS5
CVS6

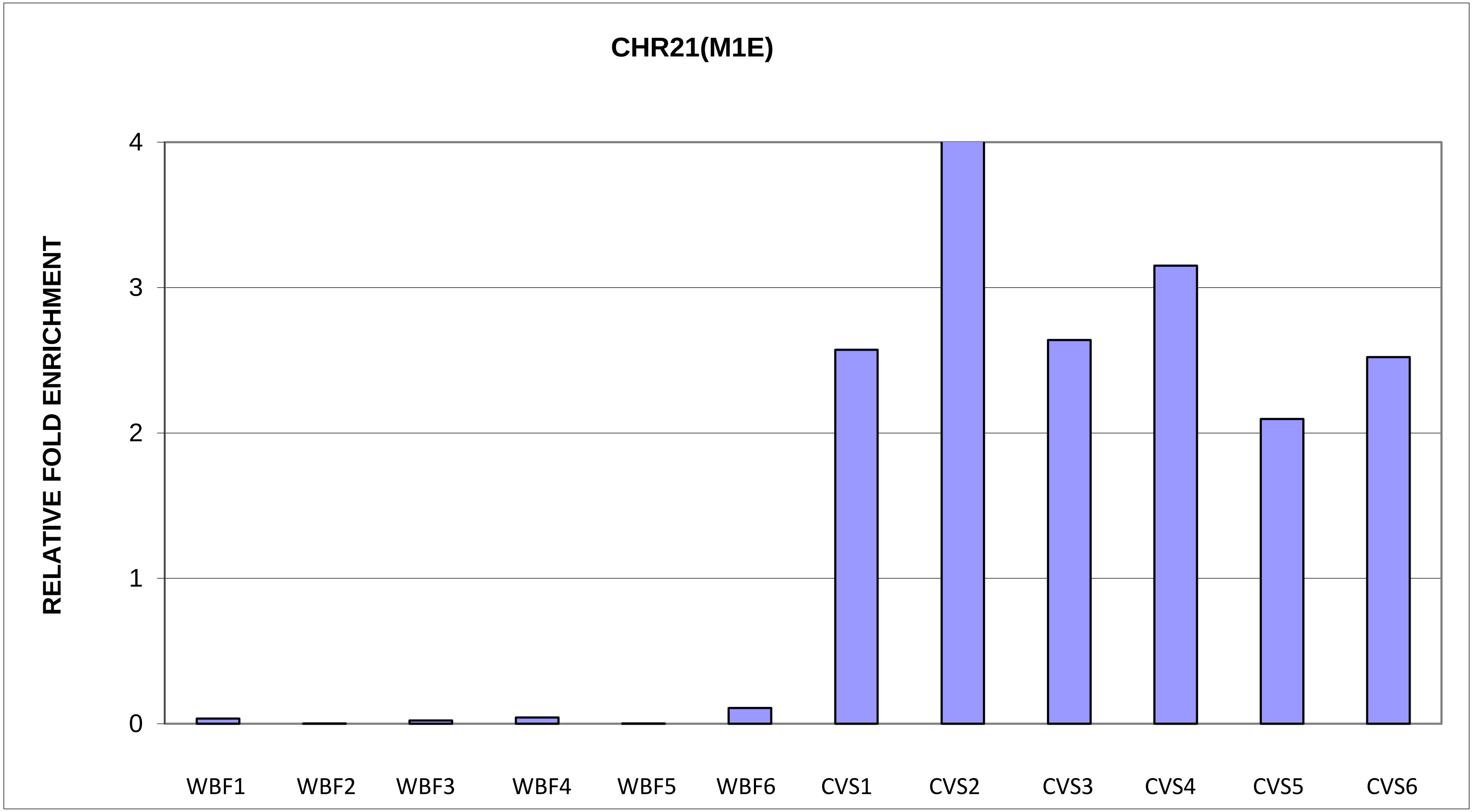

CHR21(M1E)
RELATIVE FOLD ENRICHMENT
4
3
2
1
0
WBF1
WBF2
WBF3
WBF4
WBF5
WBF6
CVS1
CVS2
CVS3
CVS4
CVS5
CVS6

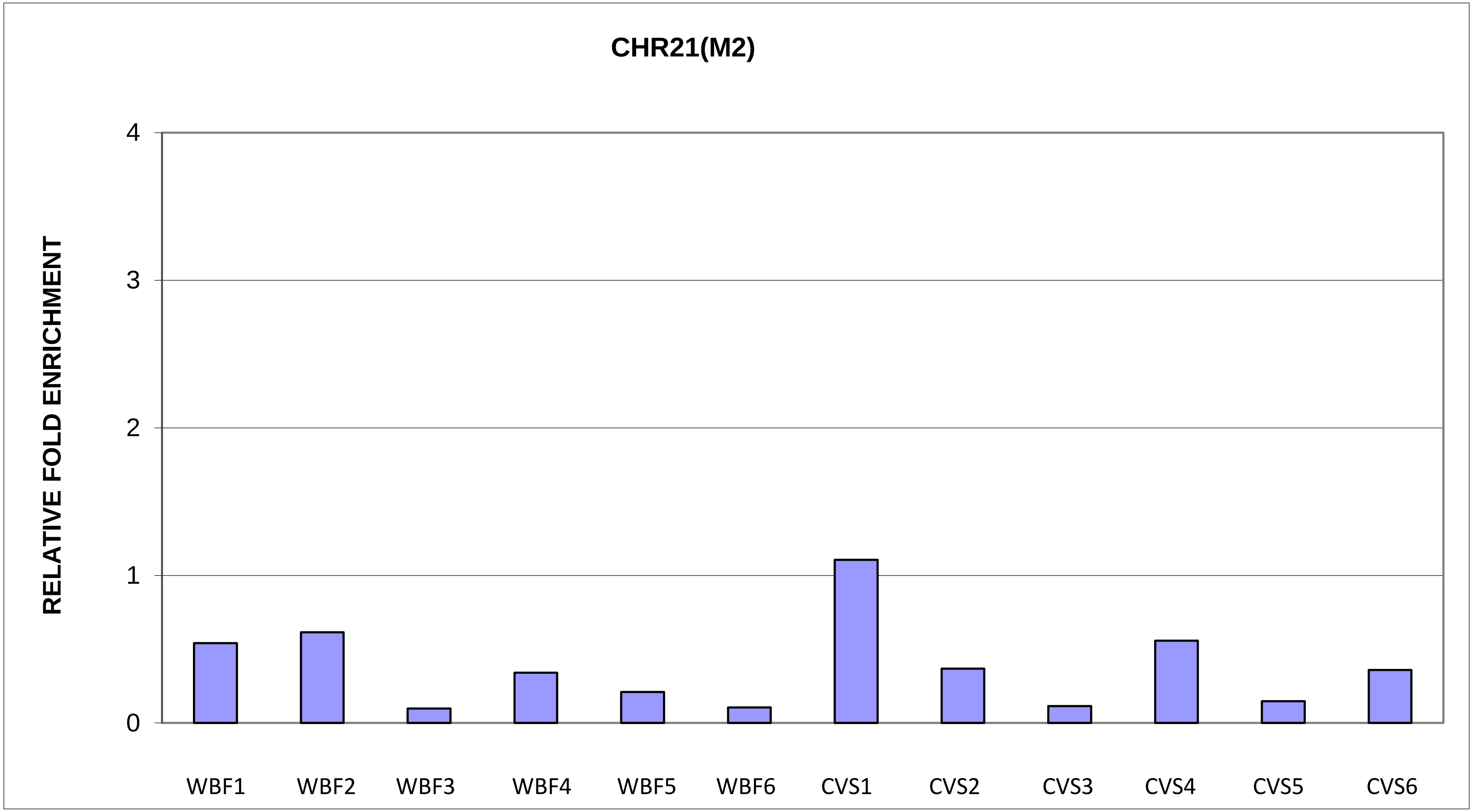
CHR21(M2)
RELATIVE FOLD ENRICHMENT
0
1
2
3
4
WBF1
WBF2
WBF3
WBF4
WBF5
WBF6
CVS1
CVS2
CVS3
CVS4
CVS5
CVS6

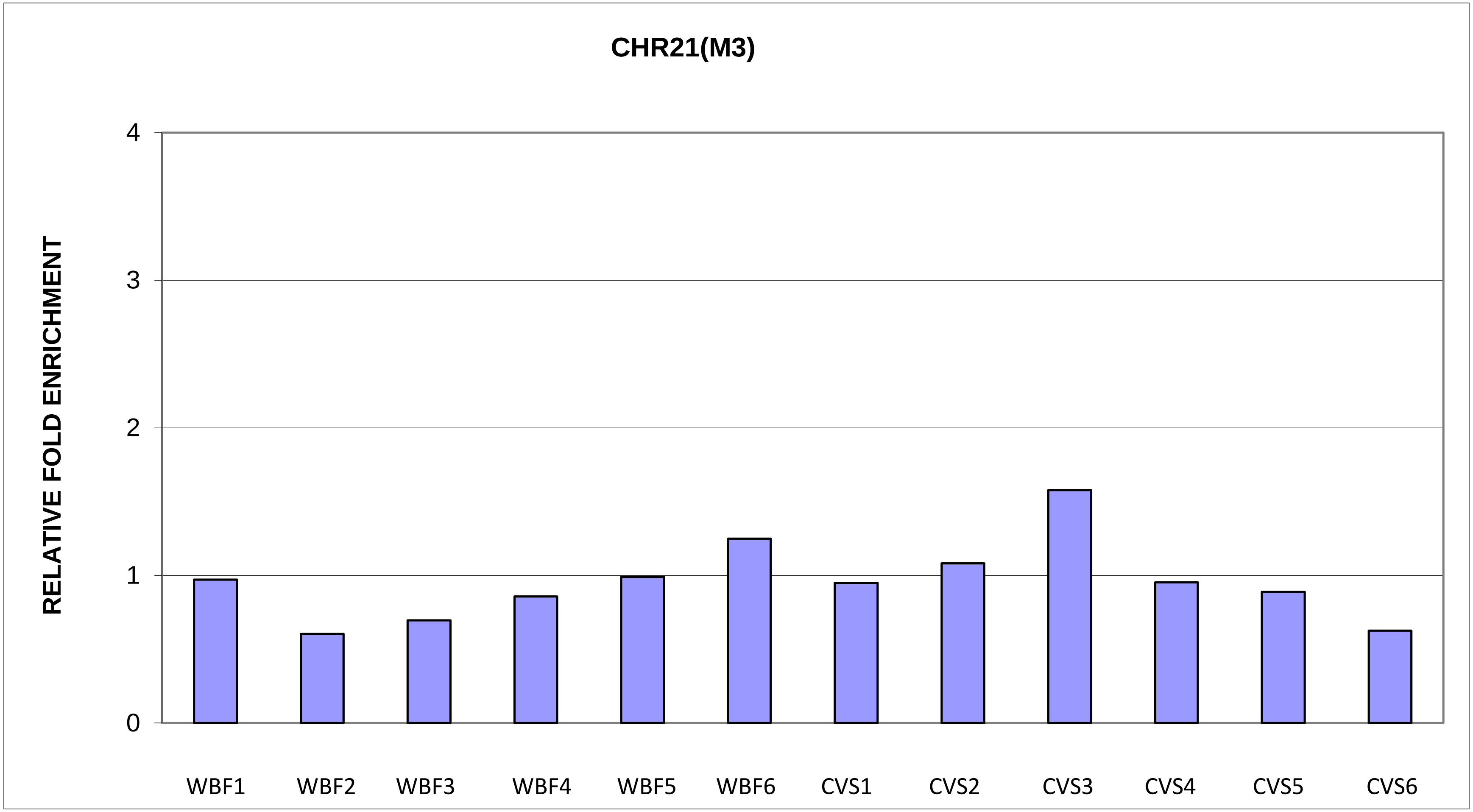
CHR21(M3)
RELATIVE FOLD ENRICHMENT
4
3
2
1
0
WBF1
WBF2
WBF3
WBF4
WBF5
WBF6
CVS1
CVS2
CVS3
CVS4
CVS5
CVS6

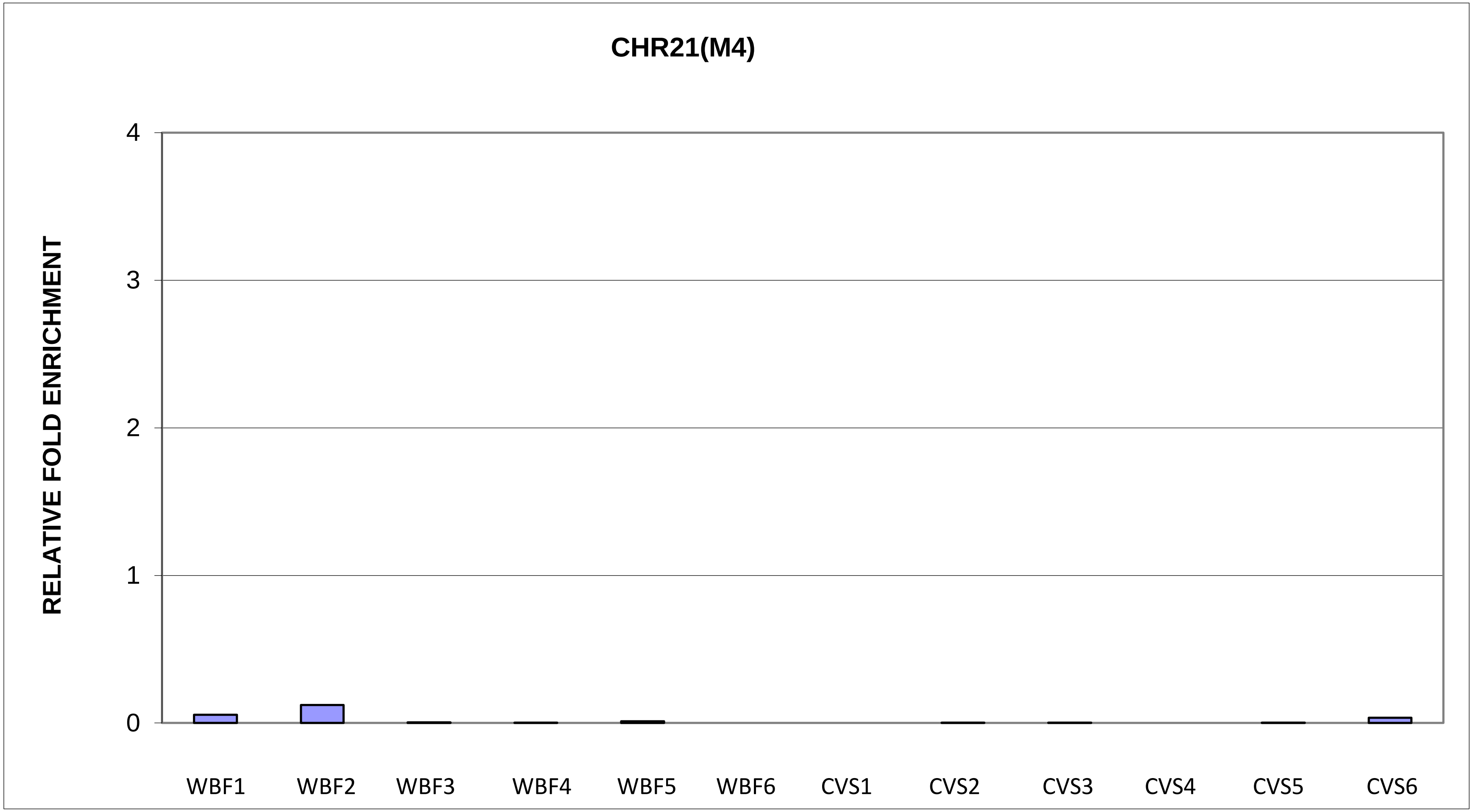

CHR21(M4)
RELATIVE FOLD ENRICHMENT
4
3
2
1
0
WBF1
WBF2
WBF3
WBF4
WBF5
WBF6
CVS1
CVS2
CVS3
CVS4
CVS5
CVS6

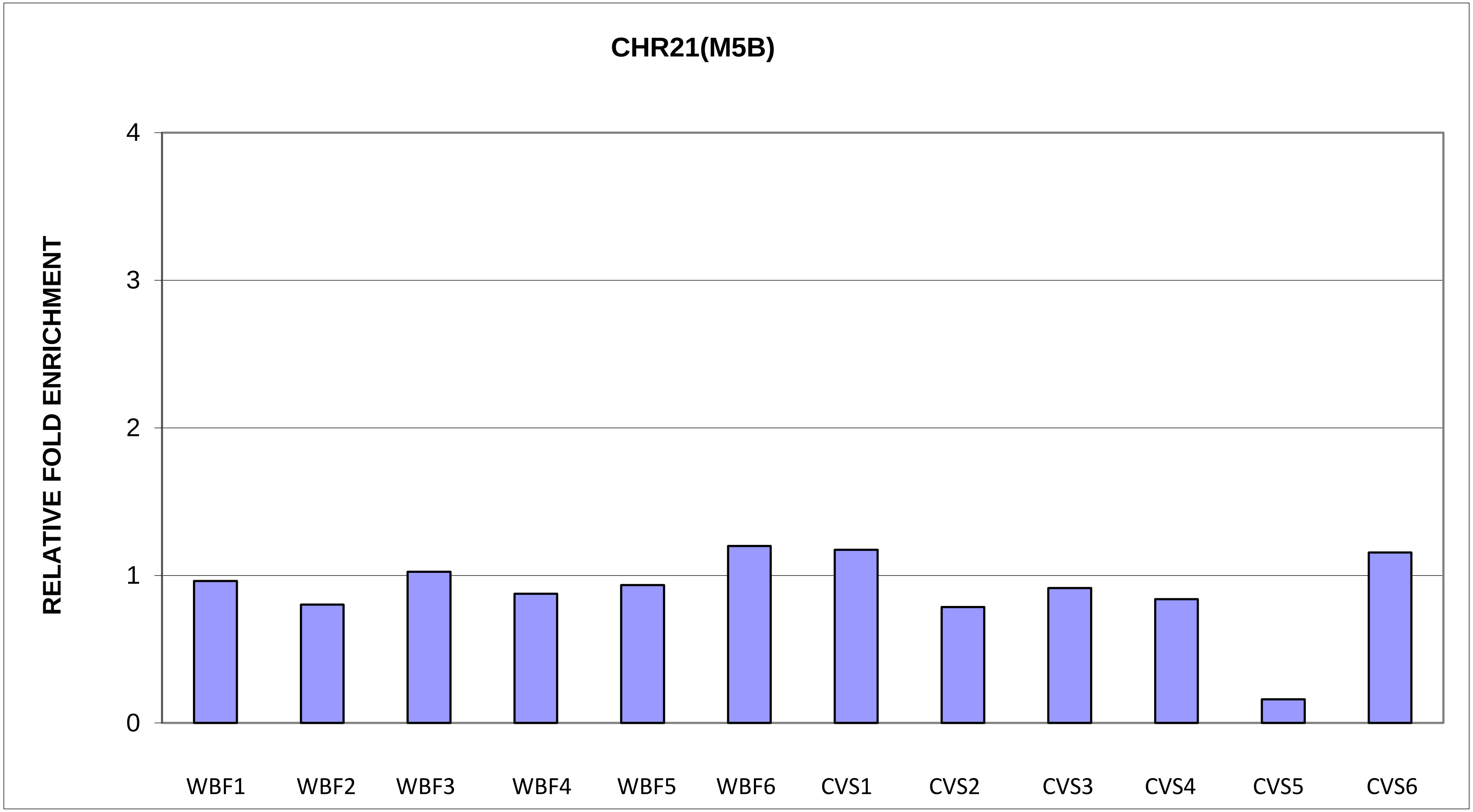
CHR21(M5B)
RELATIVE FOLD ENRICHMENT
4
3
2
1
0
WBF1
WBF2
WBF3
WBF4
WBF5
WBF6
CVS1
CVS2
CVS3
CVS4
CVS5
CVS6

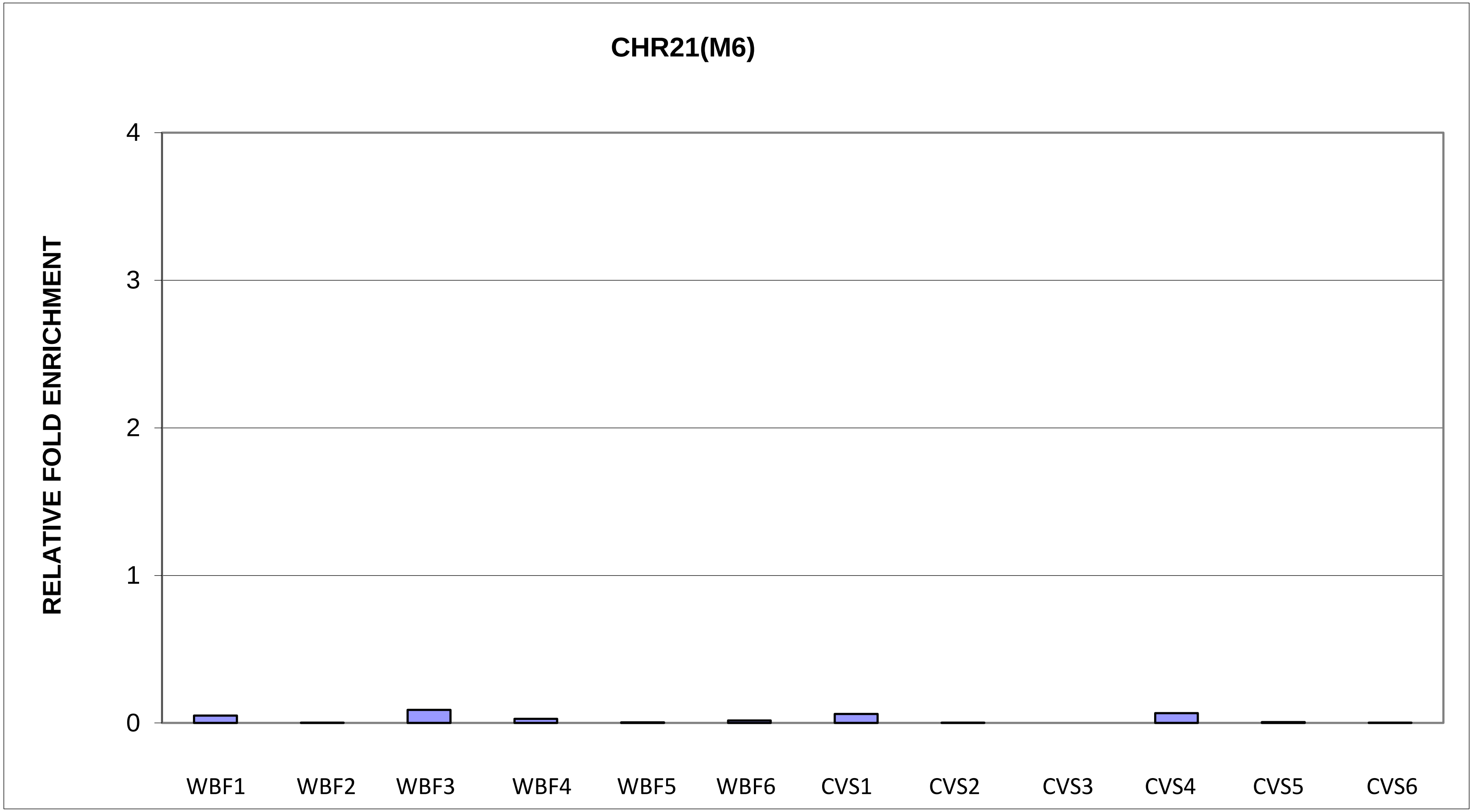

CHR21(M6)
RELATIVE FOLD ENRICHMENT
4
3
2
1
0
WBF1
WBF2
WBF3
WBF4
WBF5
WBF6
CVS1
CVS2
CVS3
CVS4
CVS5
CVS6

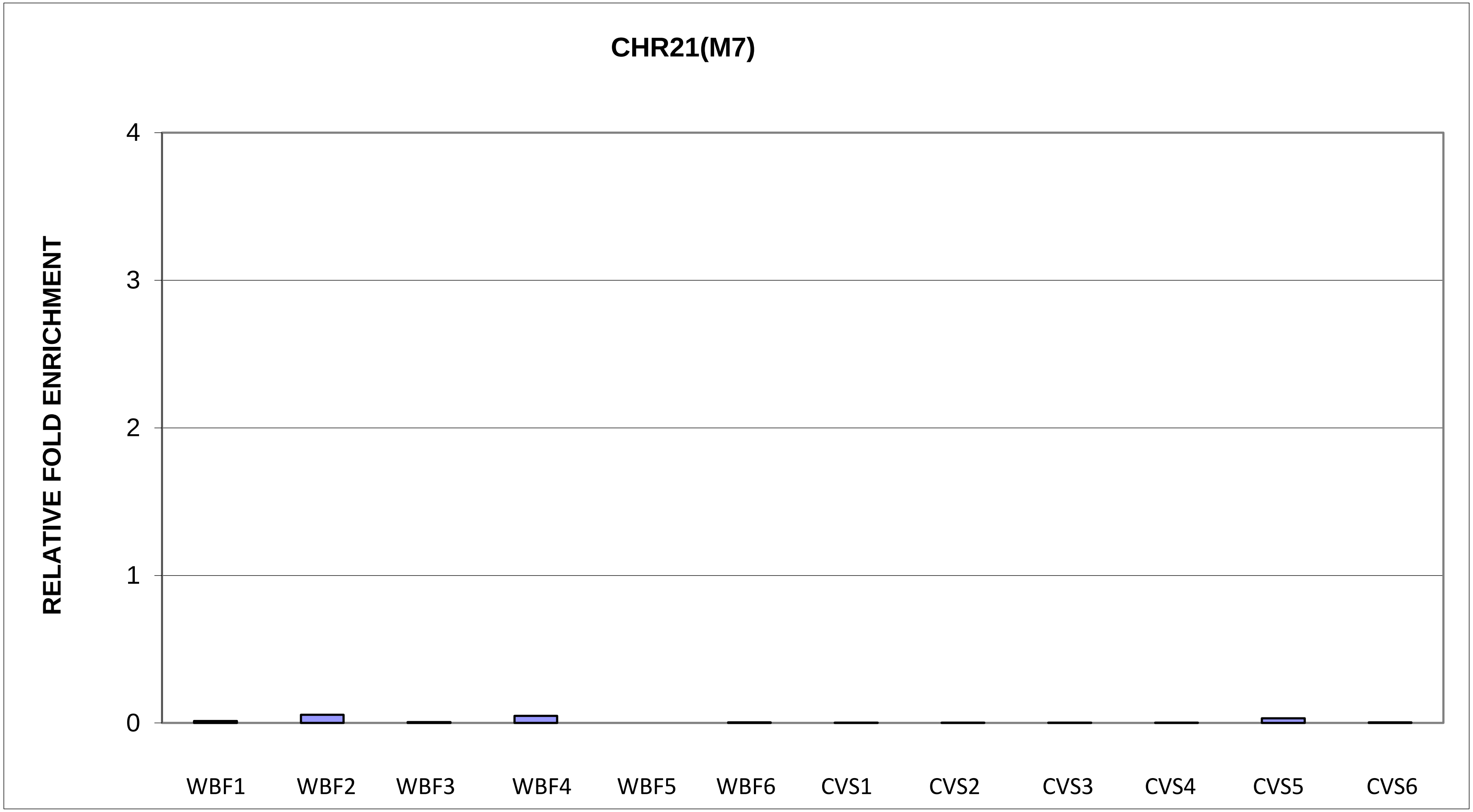
CHR21(M7)
RELATIVE FOLD ENRICHMENT
4
3
2
1
0
WBF1
WBF2
WBF3
WBF4
WBF5
WBF6
CVS1
CVS2
CVS3
CVS4
CVS5
CVS6

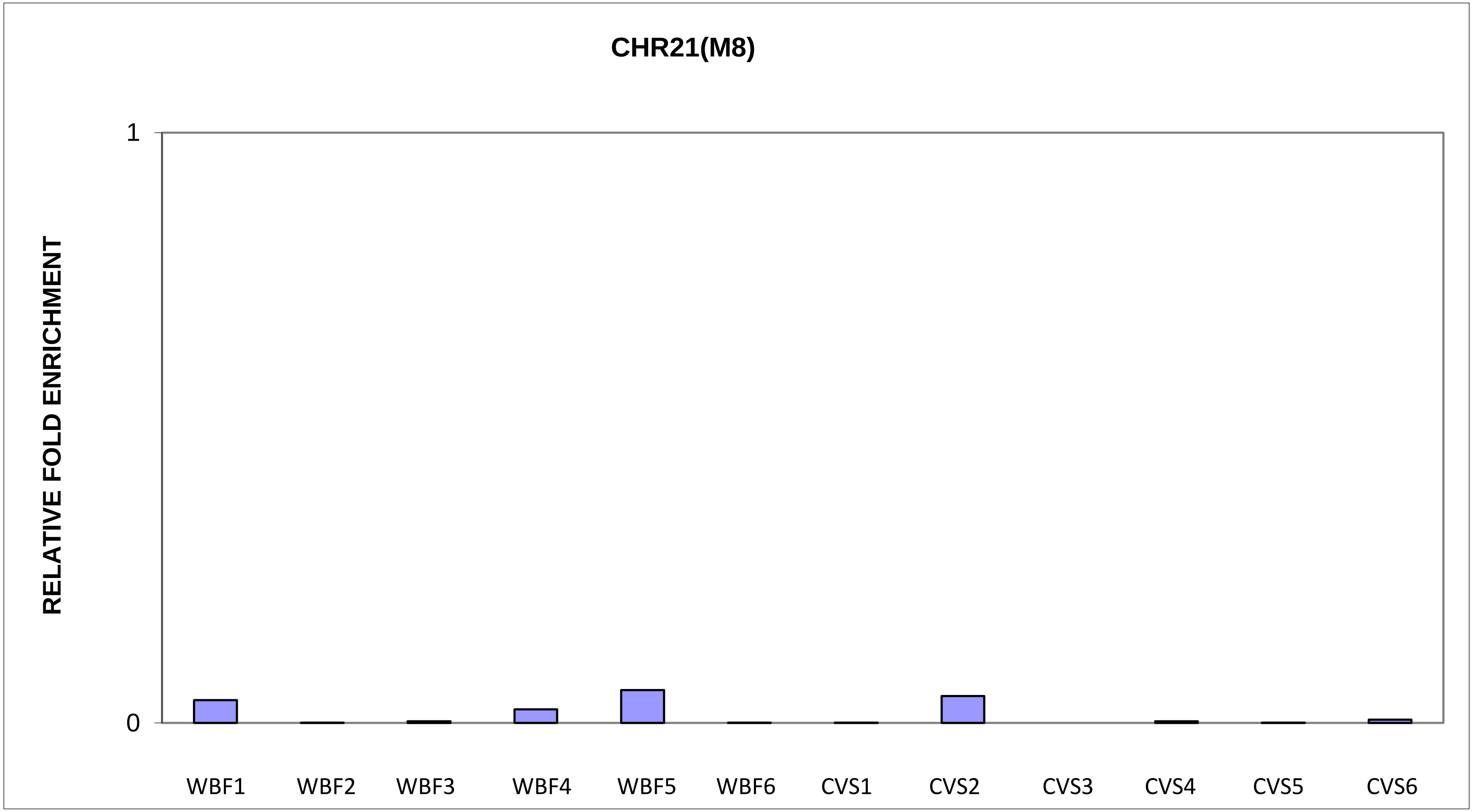
CHR21(M8)
RELATIVE FOLD ENRICHMENT
1
0
WBF1
WBF2
WBF3
WBF4
WBF5
WBF6
CVS1
CVS2
CVS3
CVS4
CVS5
CVS6

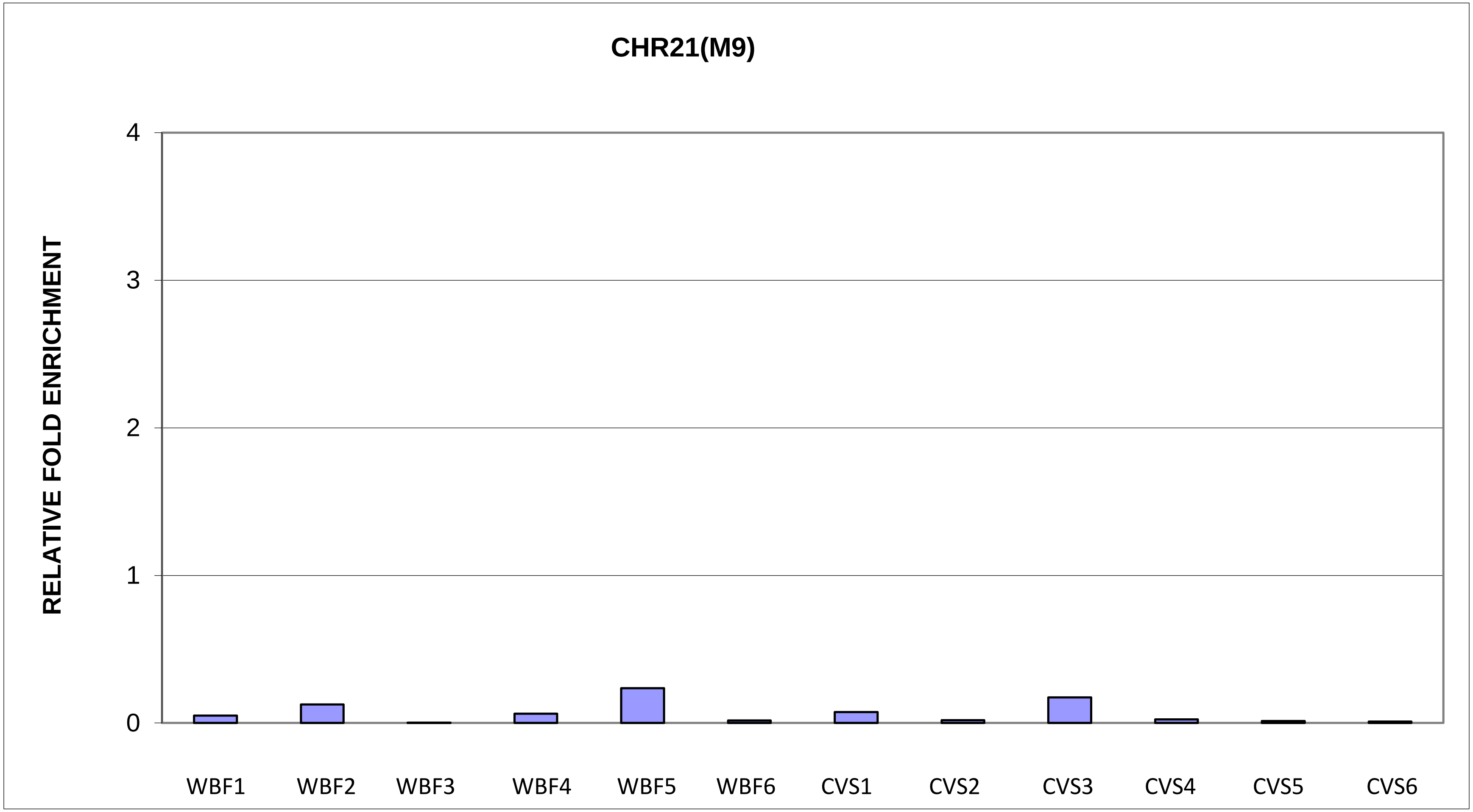
CHR21(M9)
RELATIVE FOLD ENRICHMENT
4
3
2
1
0
WBF1
WBF2
WBF3
WBF4
WBF5
WBF6
CVS1
CVS2
CVS3
CVS4
CVS5
CVS6

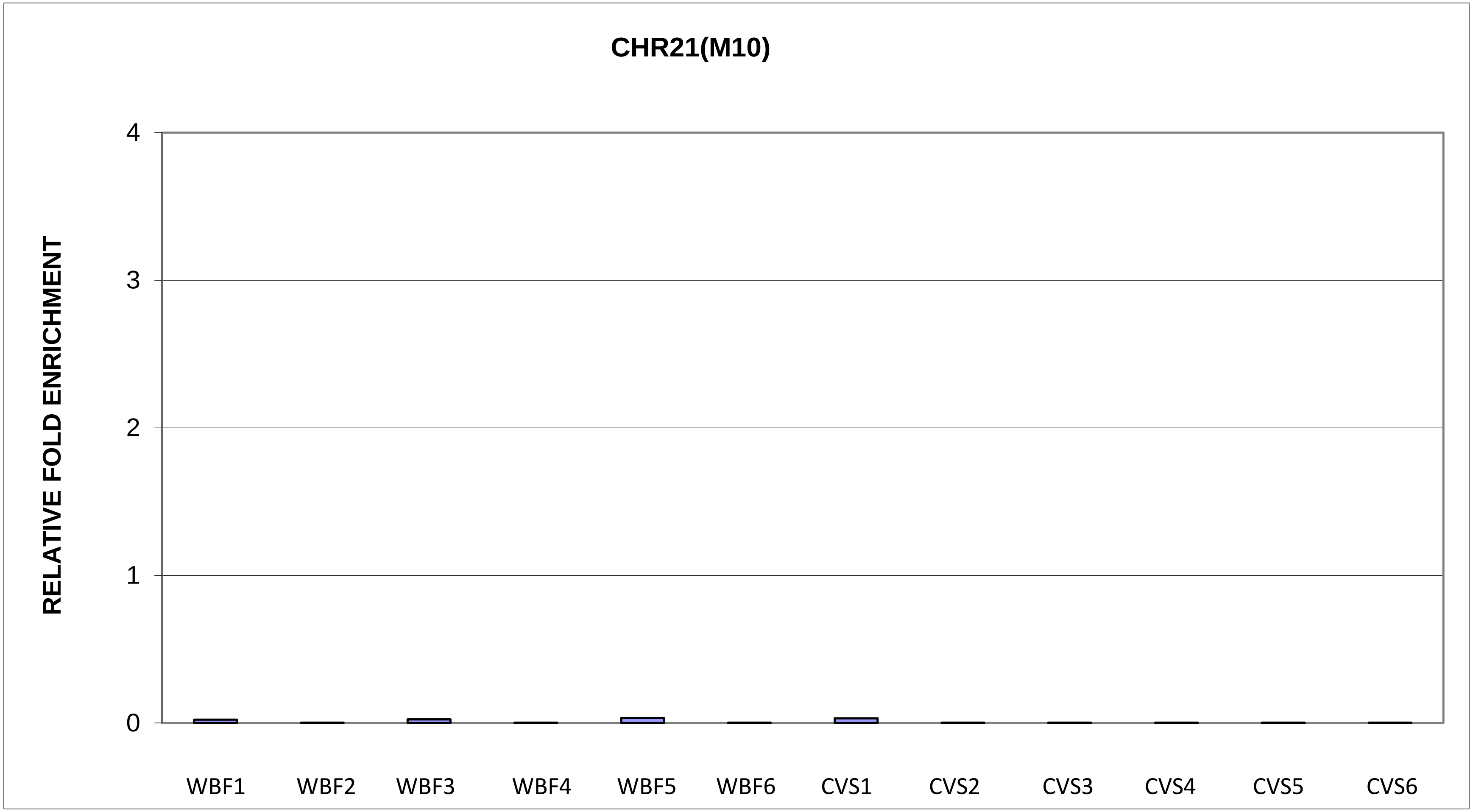
CHR21(M10)
RELATIVE FOLD ENRICHMENT
4
3
2
1
0
WBF1
WBF2
WBF3
WBF4
WBF5
WBF6
CVS1
CVS2
CVS3
CVS4
CVS5
CVS6

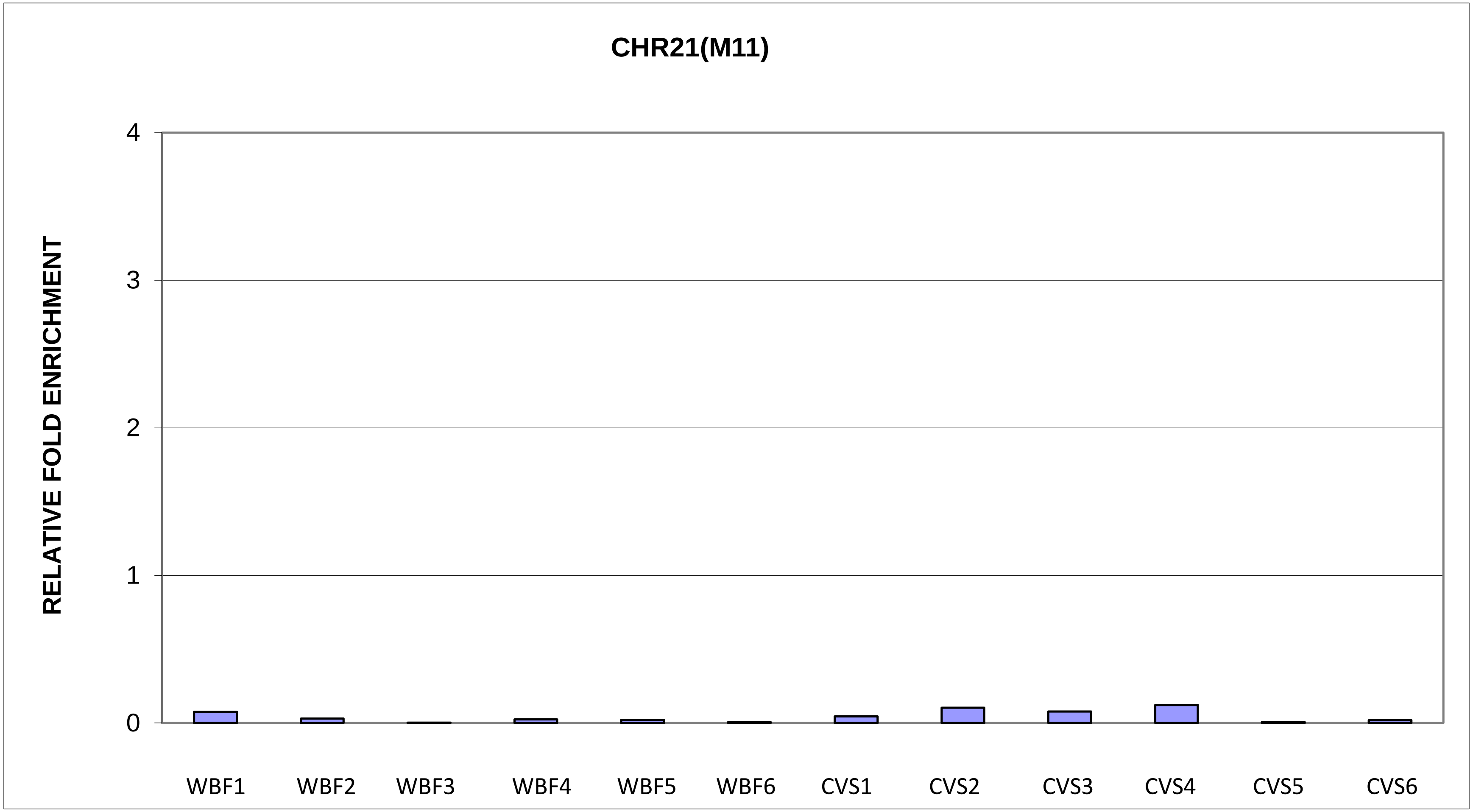

CHR21(M11)
RELATIVE FOLD ENRICHMENT
4
3
2
1
0
WBF1
WBF2
WBF3
WBF4
WBF5
WBF6
CVS1
CVS2
CVS3
CVS4
CVS5
CVS6

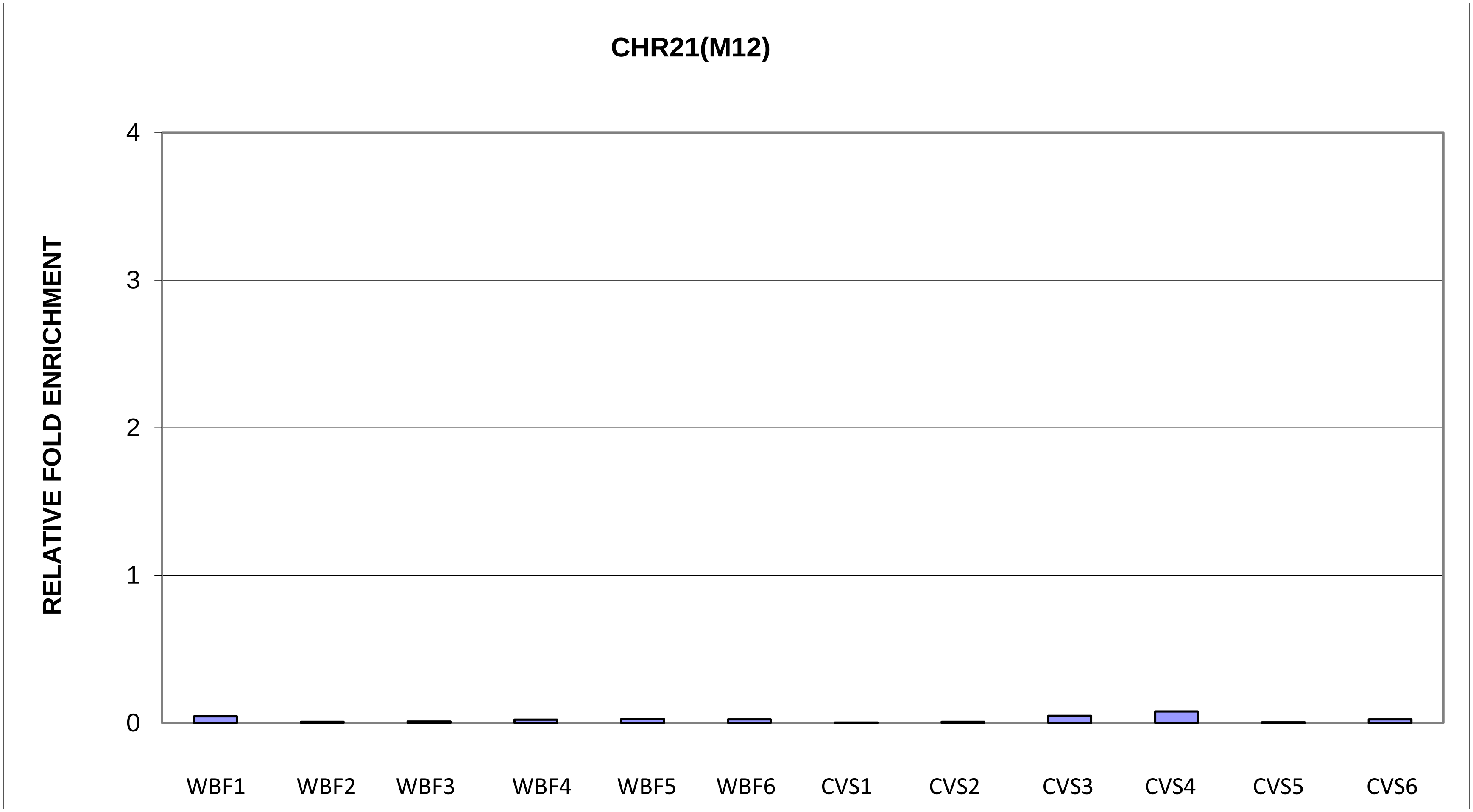
CHR21(M12)
RELATIVE FOLD ENRICHMENT
4
3
2
1
0
WBF1
WBF2
WBF3
WBF4
WBF5
WBF6
CVS1
CVS2
CVS3
CVS4
CVS5
CVS6

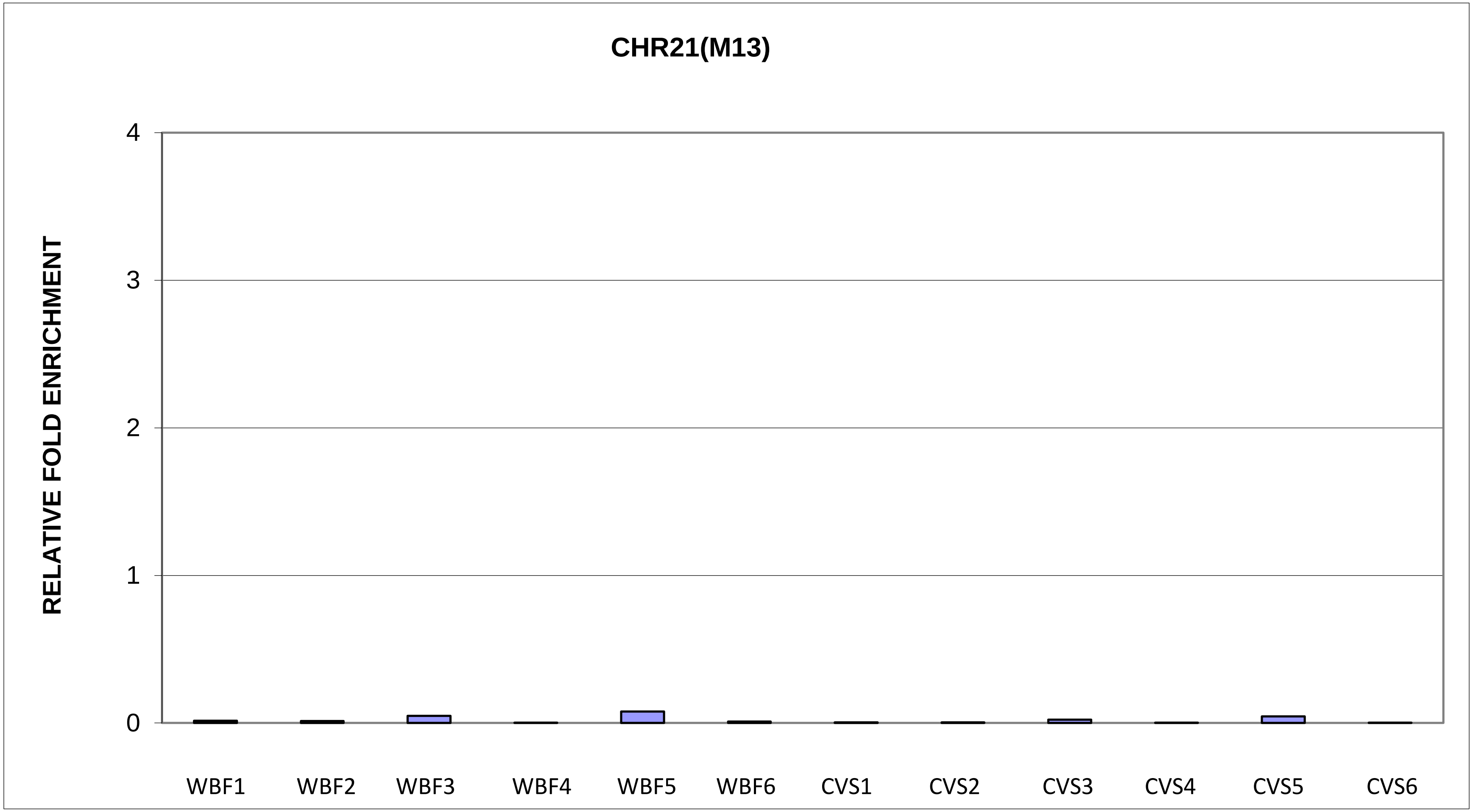
CHR21(M13)
RELATIVE FOLD ENRICHMENT
0
1
2
3
4
WBF1
WBF2
WBF3
WBF4
WBF5
WBF6
CVS1
CVS2
CVS3
CVS4
CVS5
CVS6

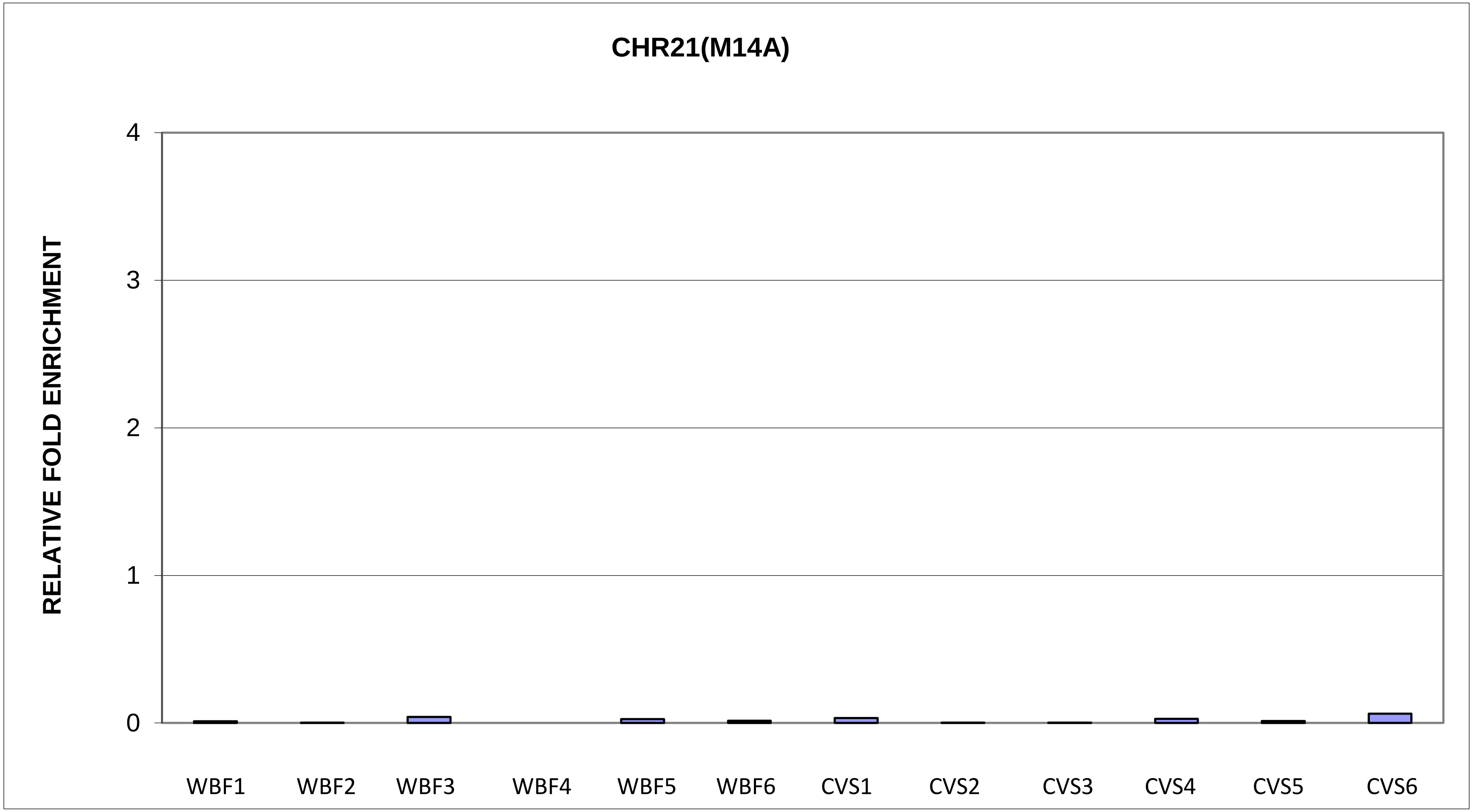
CHR21(M14A)
RELATIVE FOLD ENRICHMENT
4
3
2
1
0
WBF1
WBF2
WBF3
WBF4
WBF5
WBF6
CVS1
CVS2
CVS3
CVS4
CVS5
CVS6

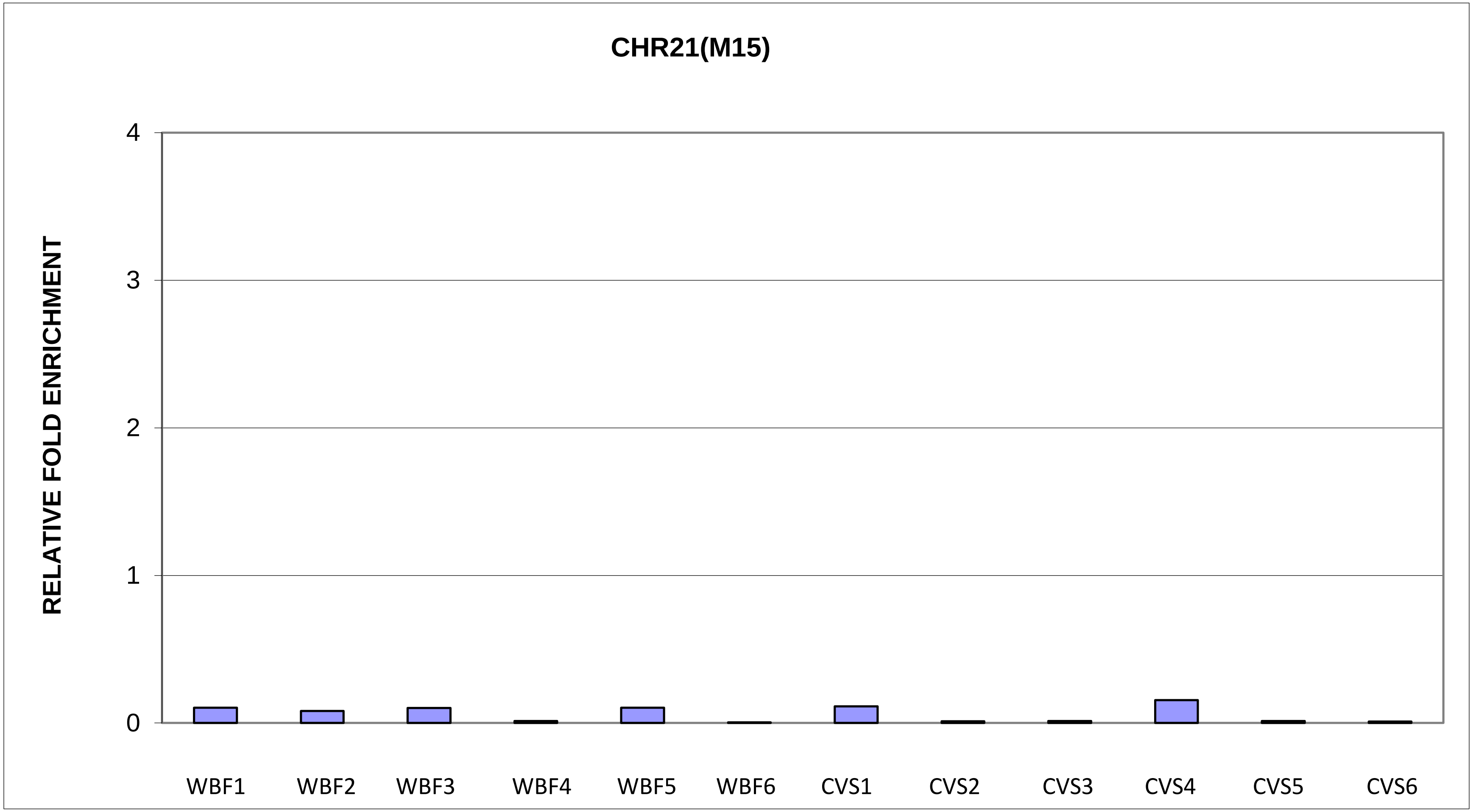
CHR21(M15)
RELATIVE FOLD ENRICHMENT
4
3
2
1
0
WBF1
WBF2
WBF3
WBF4
WBF5
WBF6
CVS1
CVS2
CVS3
CVS4
CVS5
CVS6

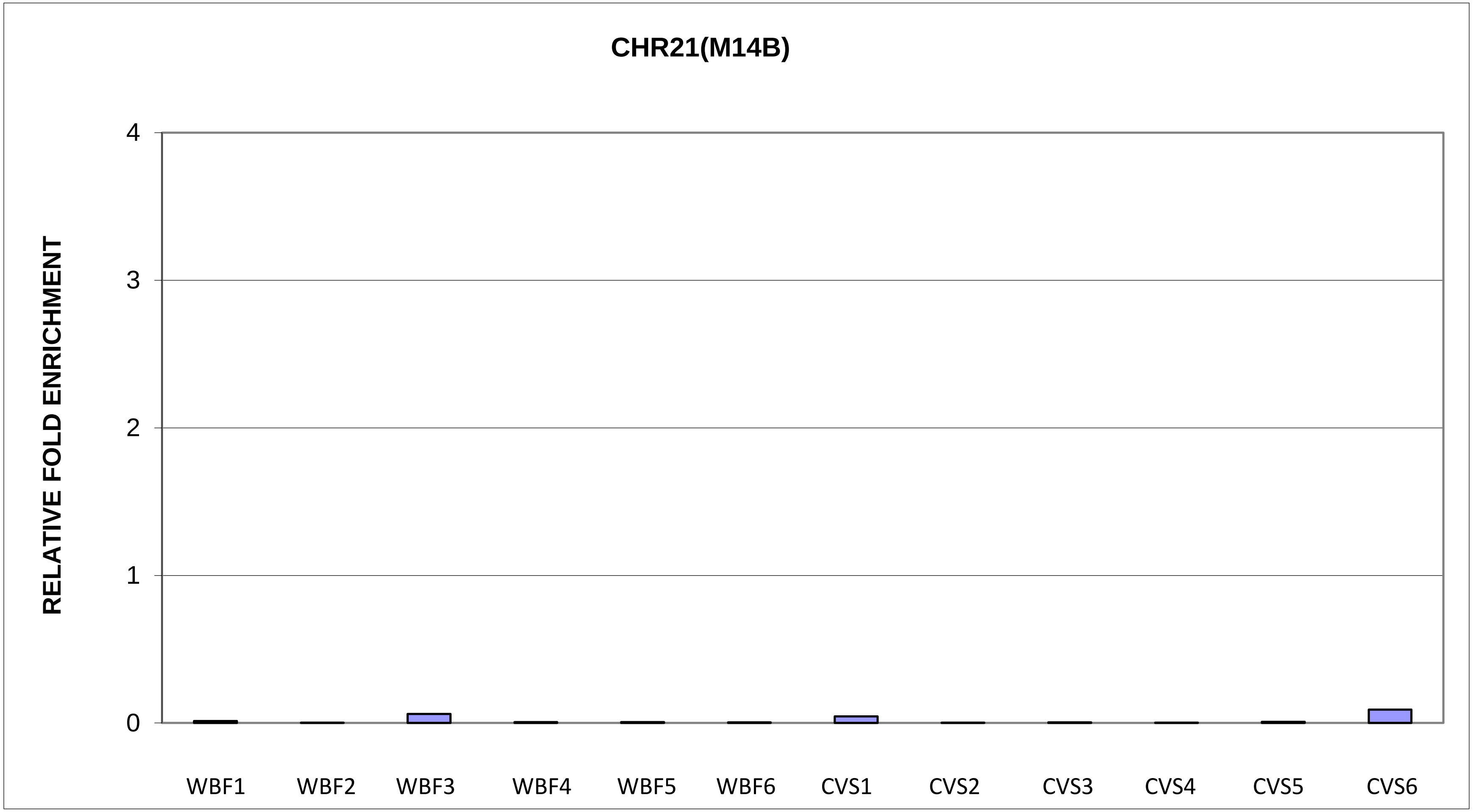
CHR21(M14B)
RELATIVE FOLD ENRICHMENT
4
3
2
1
0
WBF1
WBF2
WBF3
WBF4
WBF5
WBF6
CVS1
CVS2
CVS3
CVS4
CVS5
CVS6

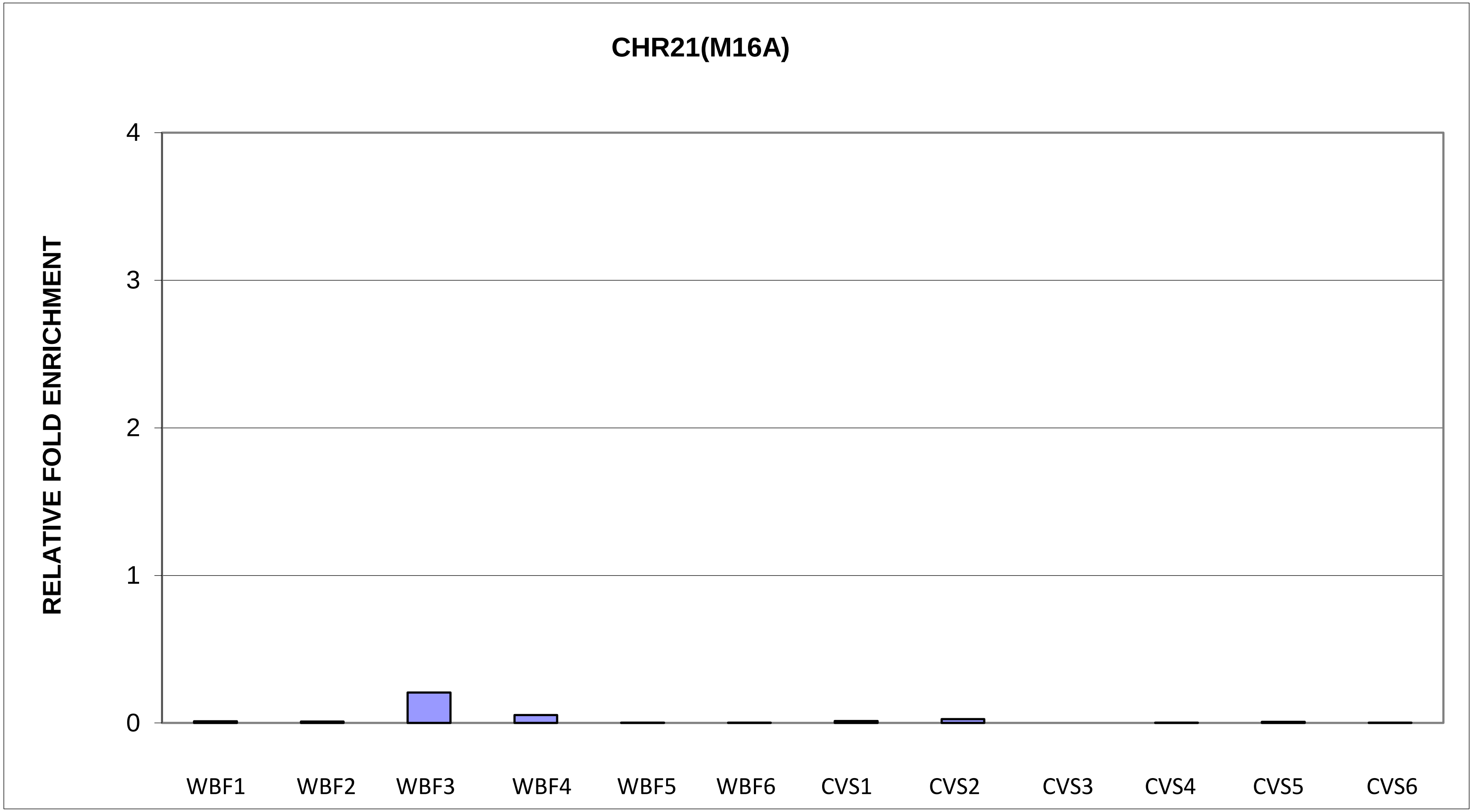
CHR21(M16A)
RELATIVE FOLD ENRICHMENT
4
3
2
1
0
WBF1
WBF2
WBF3
WBF4
WBF5
WBF6
CVS1
CVS2
CVS3
CVS4
CVS5
CVS6

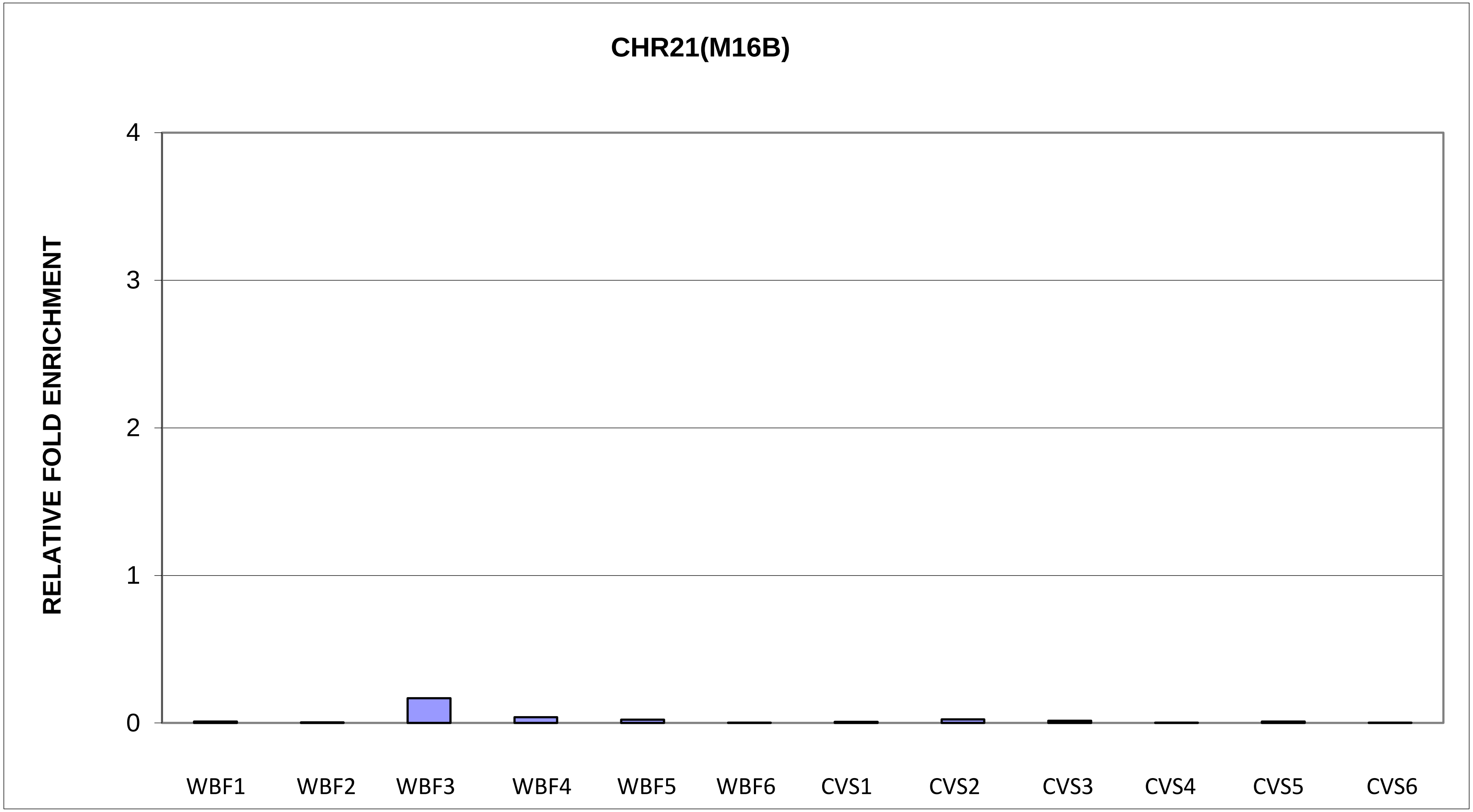
CHR21(M16B)
RELATIVE FOLD ENRICHMENT
4
3
2
1
0
WBF1
WBF2
WBF3
WBF4
WBF5
WBF6
CVS1
CVS2
CVS3
CVS4
CVS5
CVS6

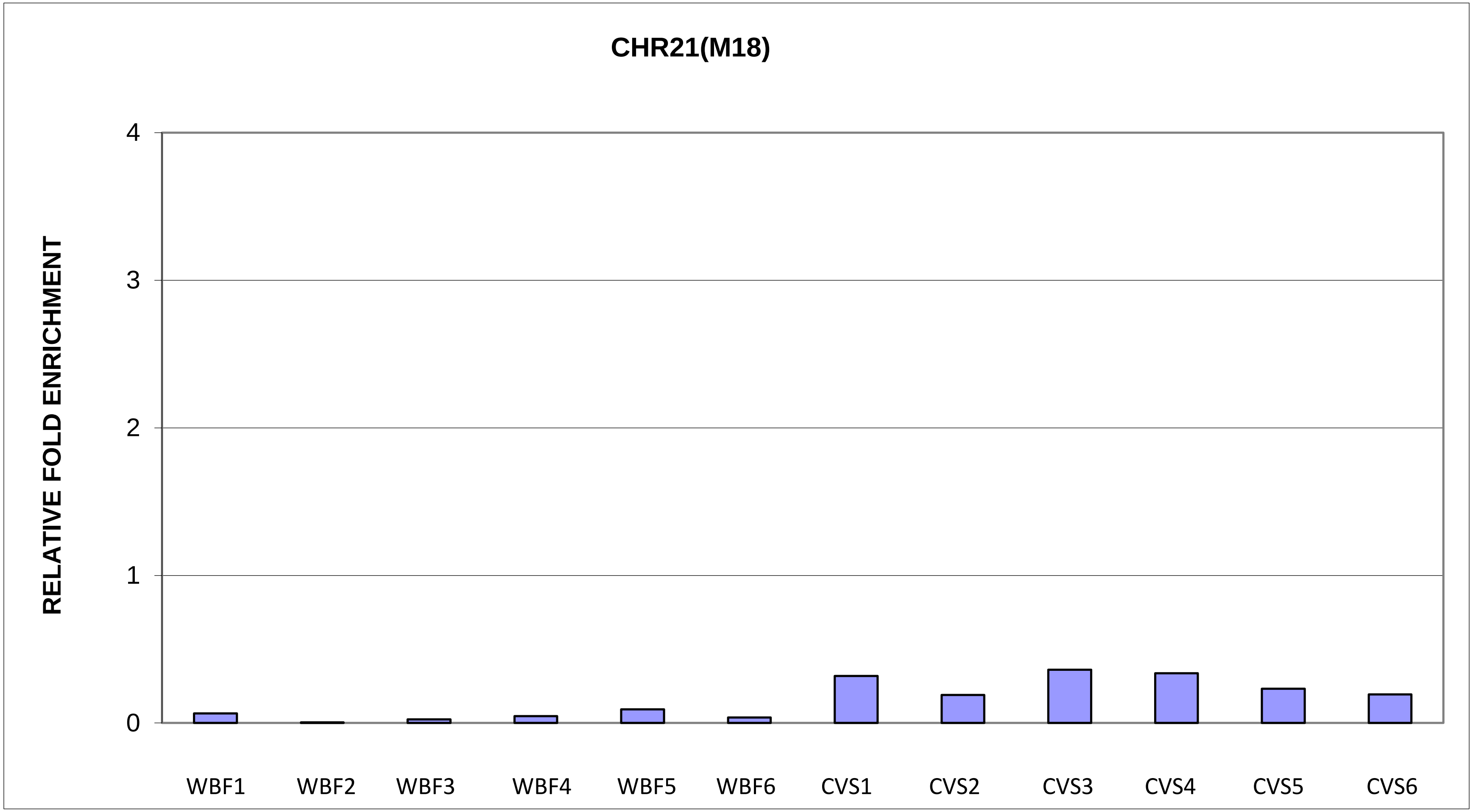
CHR21(M18)
RELATIVE FOLD ENRICHMENT
4
3
2
1
0
WBF1
WBF2
WBF3
WBF4
WBF5
WBF6
CVS1
CVS2
CVS3
CVS4
CVS5
CVS6

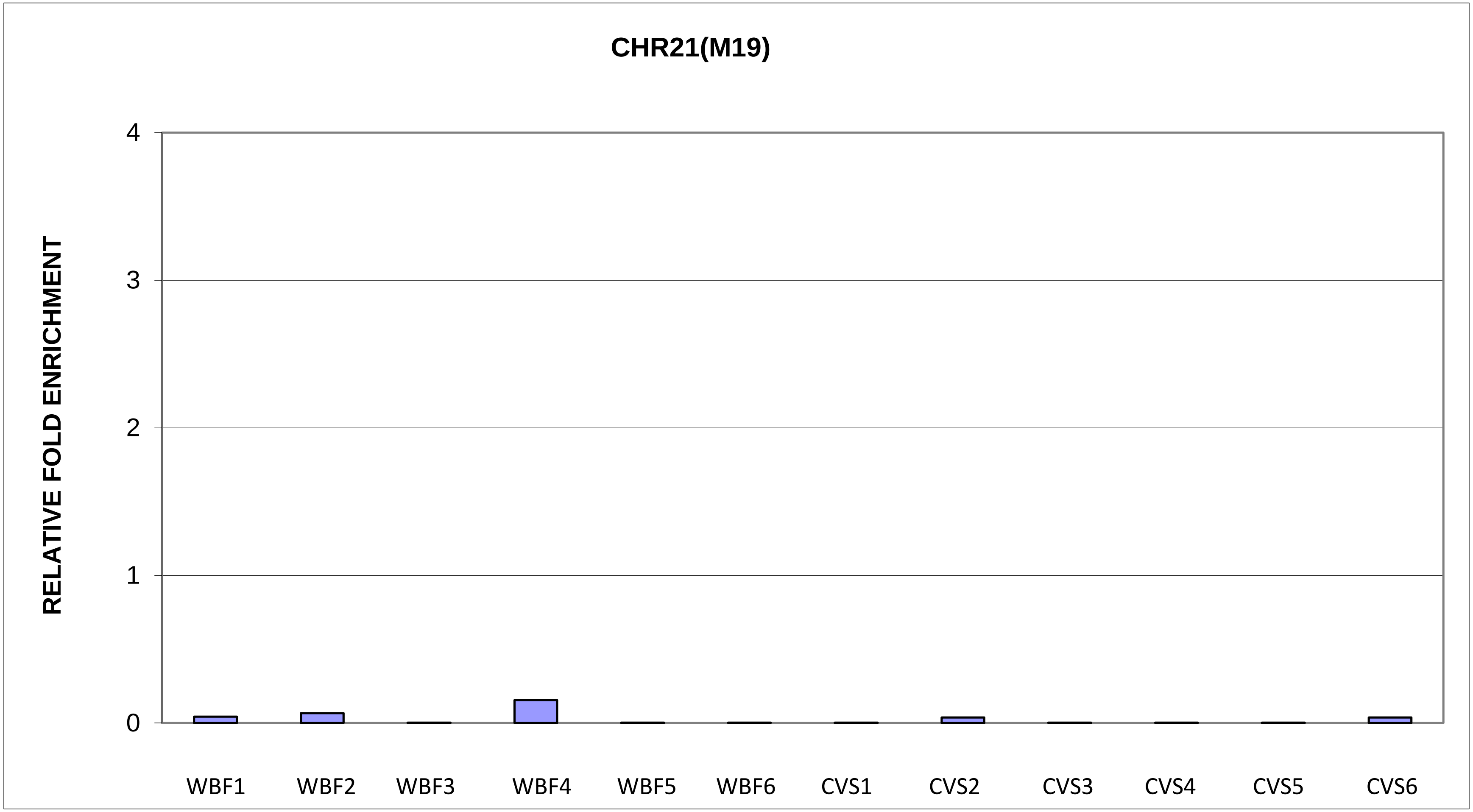
CHR21(M19)
RELATIVE FOLD ENRICHMENT
4
3
2
1
0
WBF1
WBF2
WBF3
WBF4
WBF5
WBF6
CVS1
CVS2
CVS3
CVS4
CVS5
CVS6

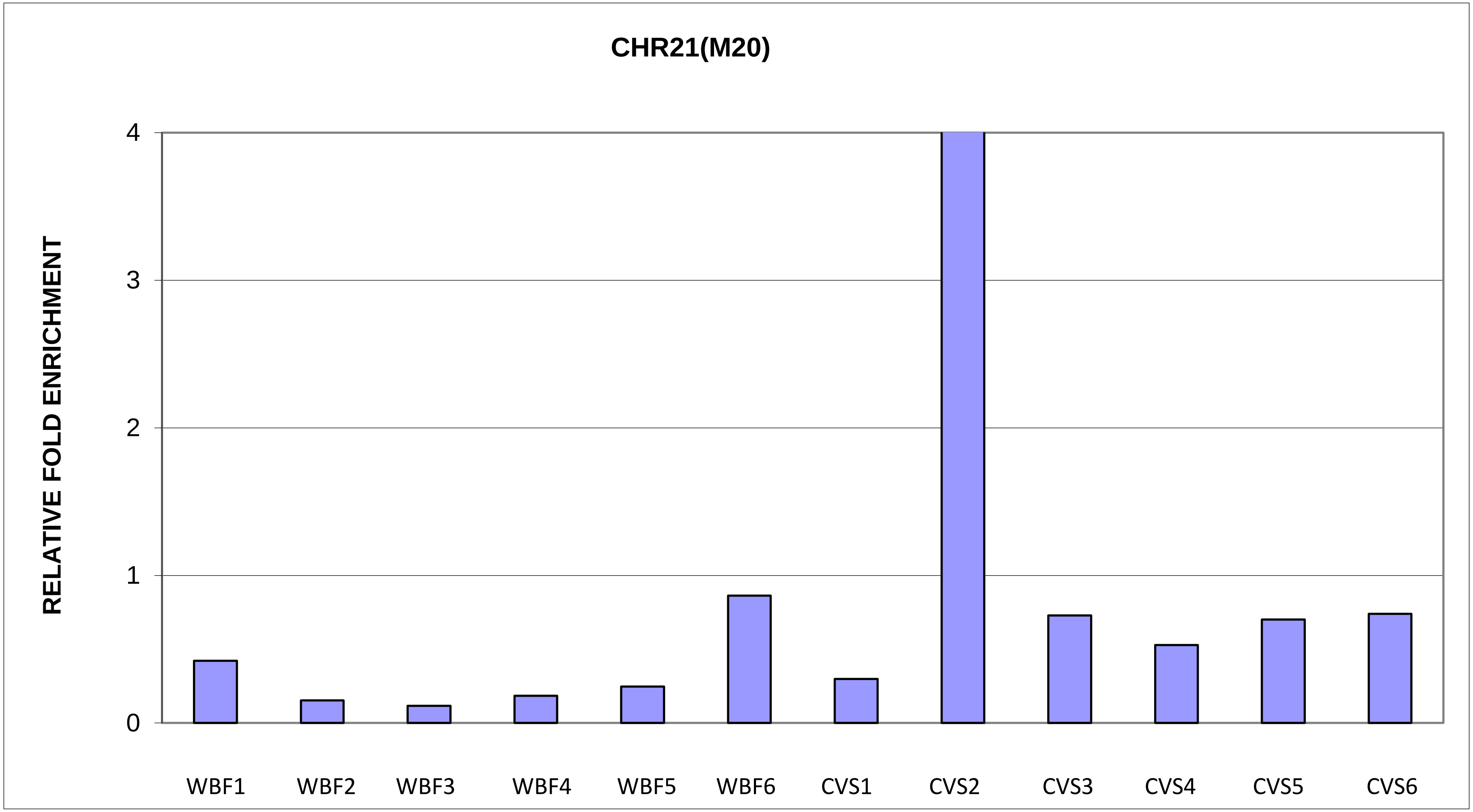

CHR21(M20)
RELATIVE FOLD ENRICHMENT
4
3
2
1
0
WBF1
WBF2
WBF3
WBF4
WBF5
WBF6
CVS1
CVS2
CVS3
CVS4
CVS5
CVS6

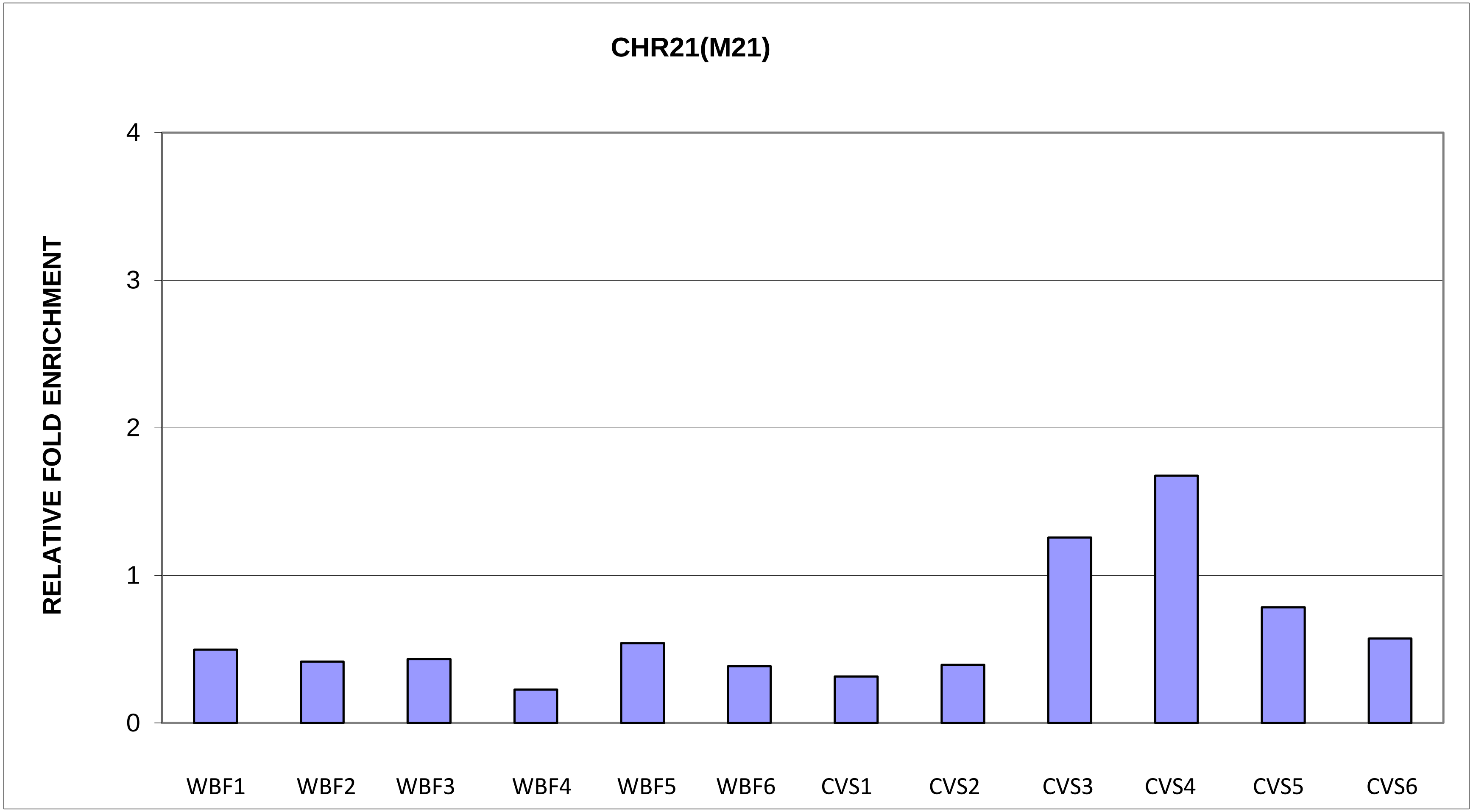
CHR21(M21)
RELATIVE FOLD ENRICHMENT
4
3
2
1
0
WBF1
WBF2
WBF3
WBF4
WBF5
WBF6
CVS1
CVS2
CVS3
CVS4
CVS5
CVS6

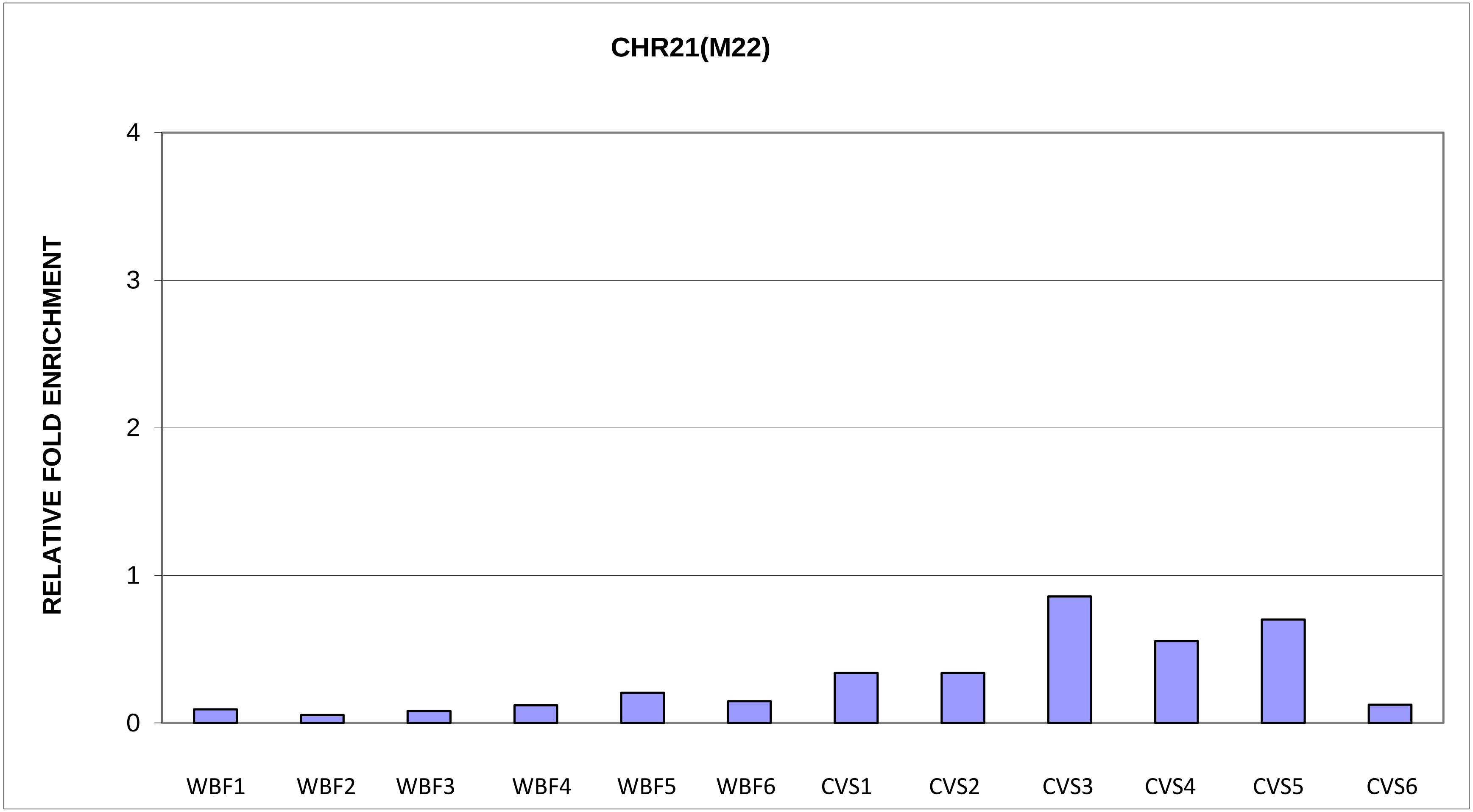

CHR21(M22)
RELATIVE FOLD ENRICHMENT
0
1
2
3
4
WBF1
WBF2
WBF3
WBF4
WBF5
WBF6
CVS1
CVS2
CVS3
CVS4
CVS5
CVS6

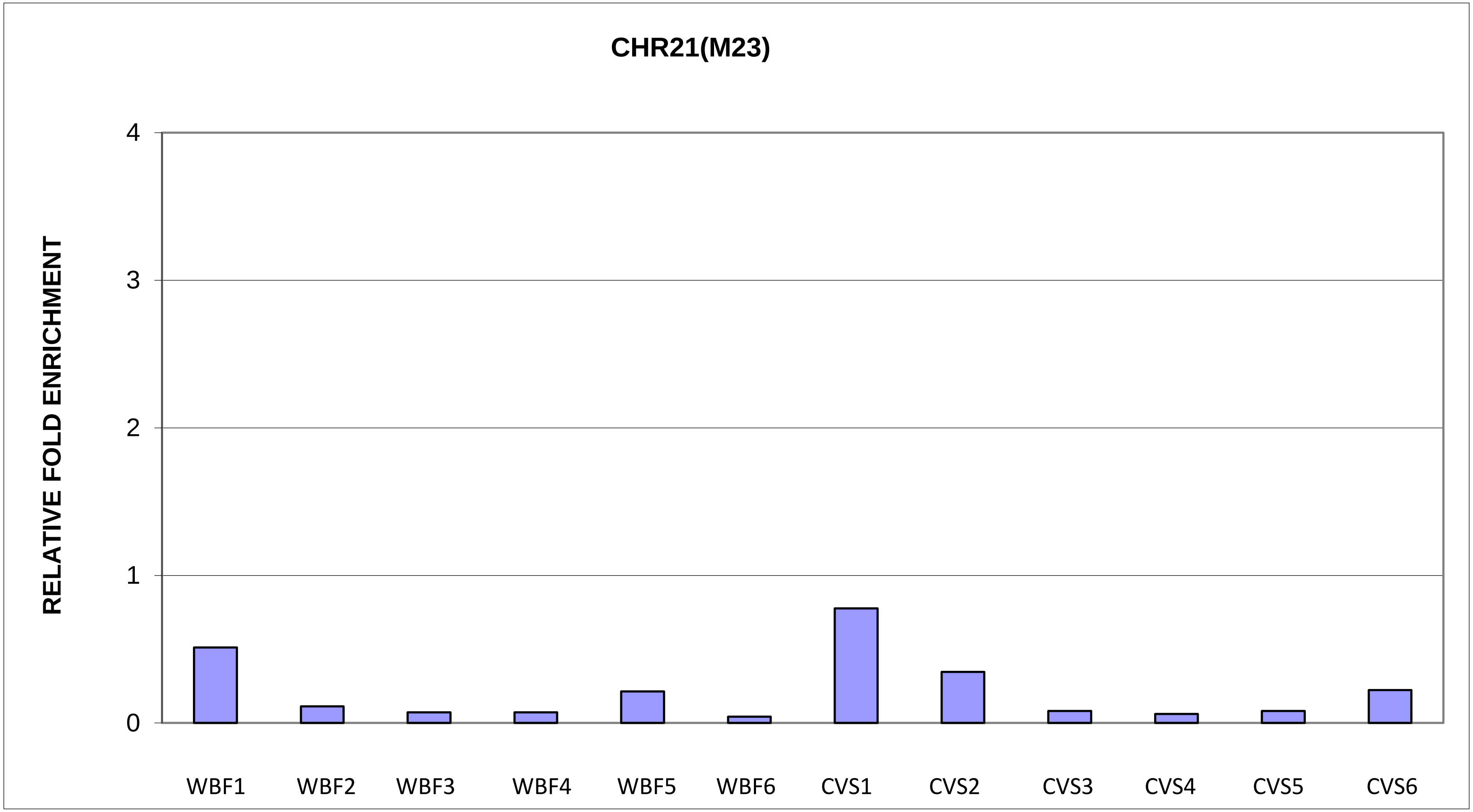
CHR21(M23)
RELATIVE FOLD ENRICHMENT
4
3
2
1
0
WBF1
WBF2
WBF3
WBF4
WBF5
WBF6
CVS1
CVS2
CVS3
CVS4
CVS5
CVS6

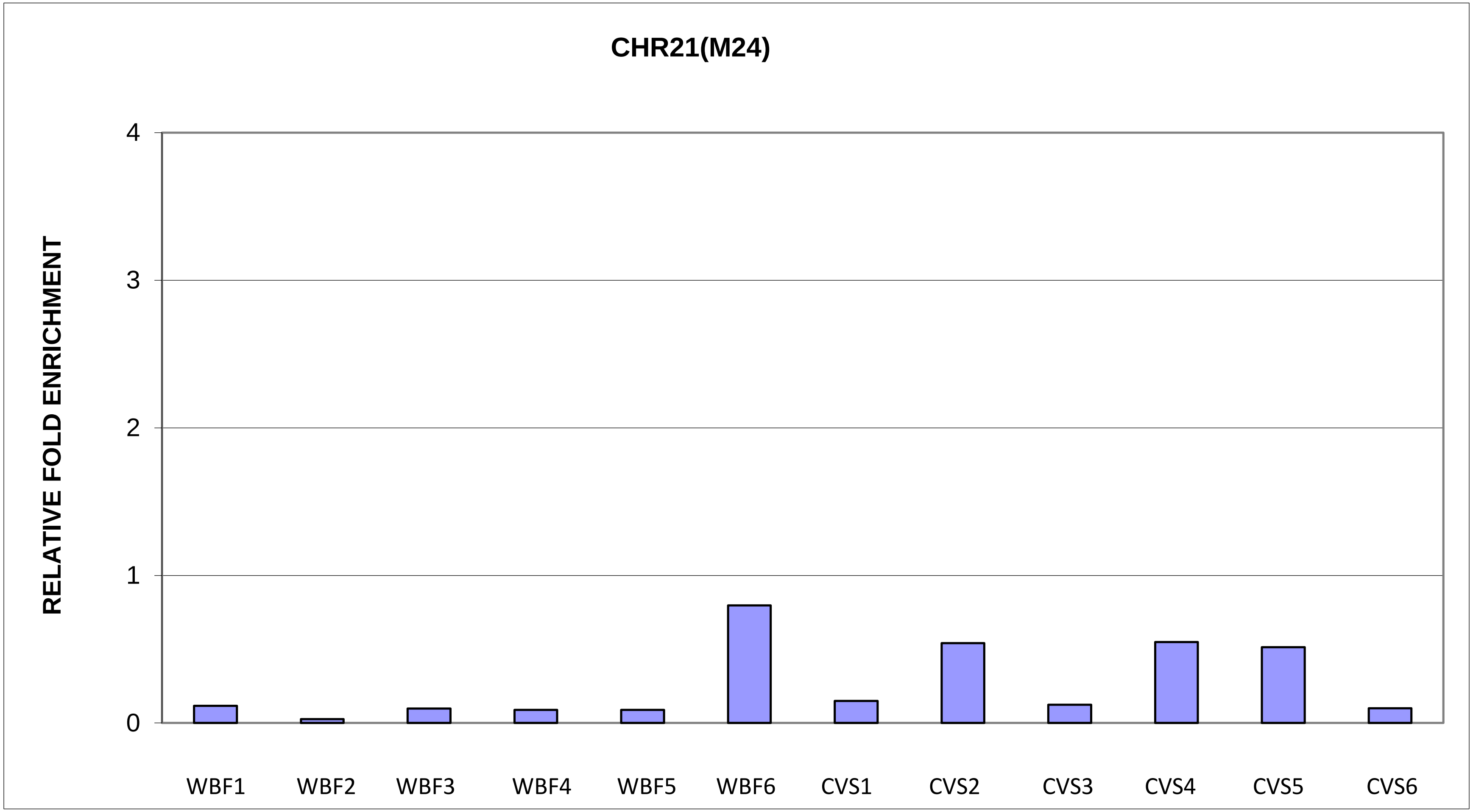
CHR21(M24)
RELATIVE FOLD ENRICHMENT
4
3
2
1
0
WBF1
WBF2
WBF3
WBF4
WBF5
WBF6
CVS1
CVS2
CVS3
CVS4
CVS5
CVS6

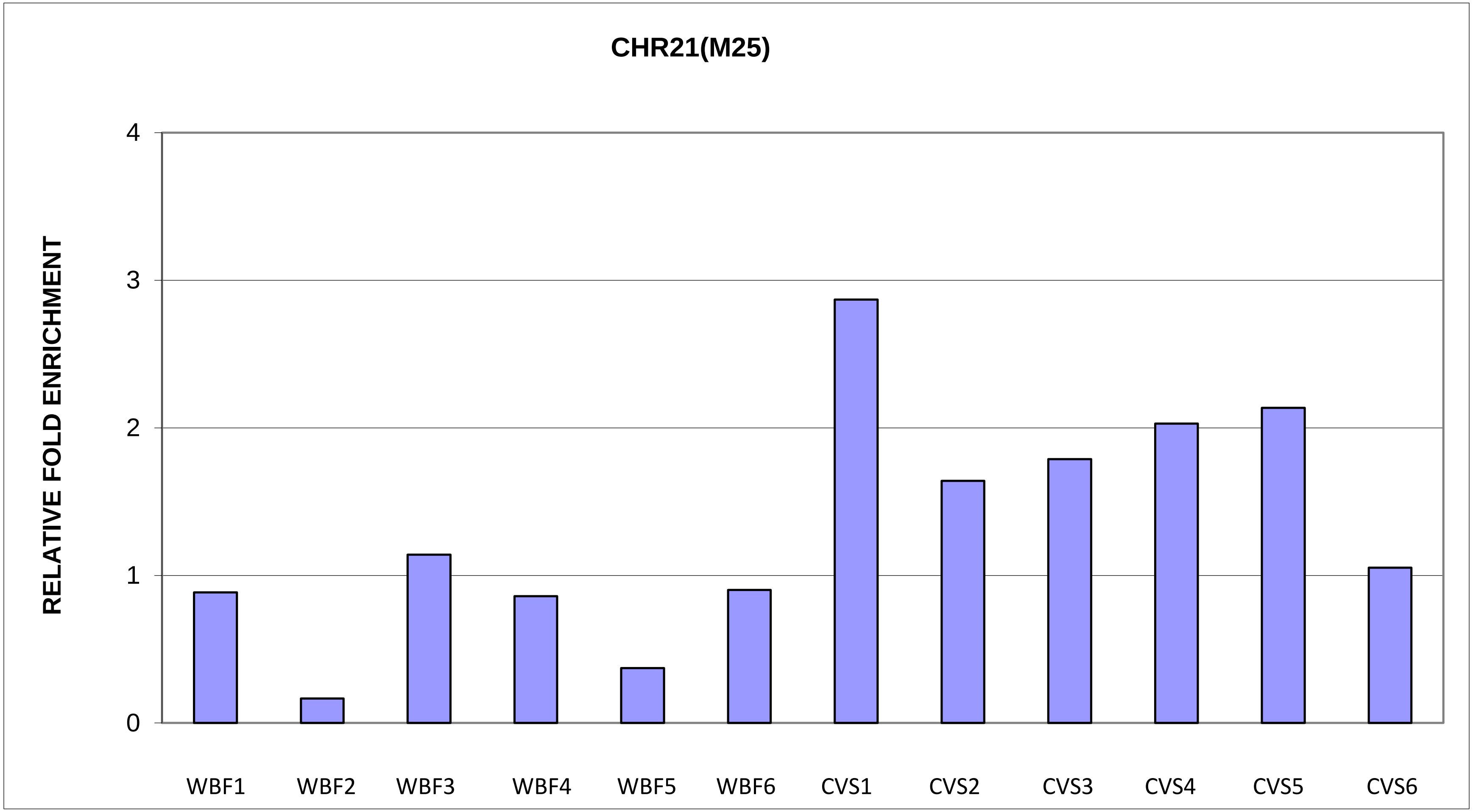

CHR21(M25)
RELATIVE FOLD ENRICHMENT
4
3
2
1
0
WBF1
WBF2
WBF3
WBF4
WBF5
WBF6
CVS1
CVS2
CVS3
CVS4
CVS5
CVS6

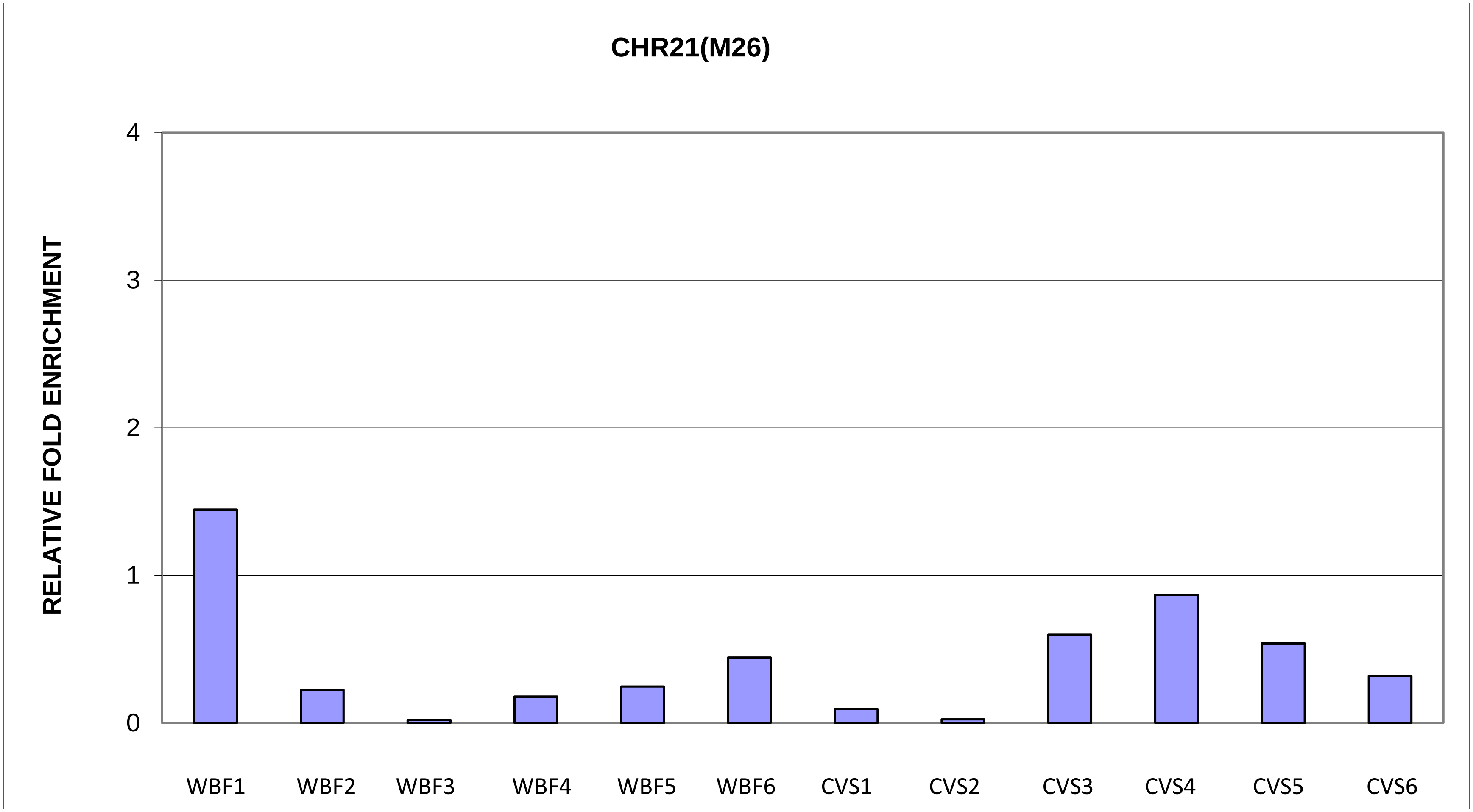

CHR21(M26)
RELATIVE FOLD ENRICHMENT
4
3
2
1
0
WBF1
WBF2
WBF3
WBF4
WBF5
WBF6
CVS1
CVS2
CVS3
CVS4
CVS5
CVS6

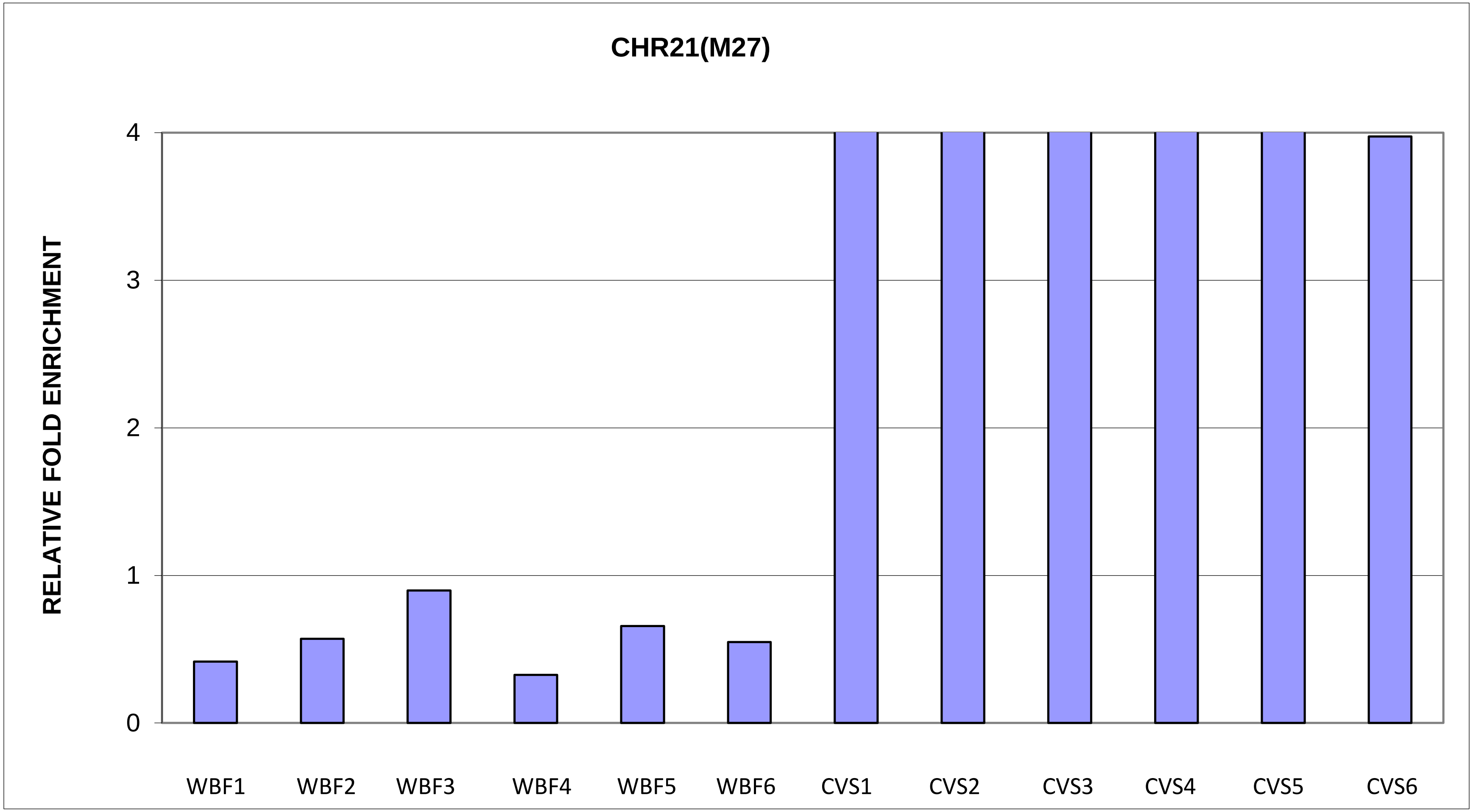

CHR21(M27)
RELATIVE FOLD ENRICHMENT
4
3
2
1
0
WBF1
WBF2
WBF3
WBF4
WBF5
WBF6
CVS1
CVS2
CVS3
CVS4
CVS5
CVS6

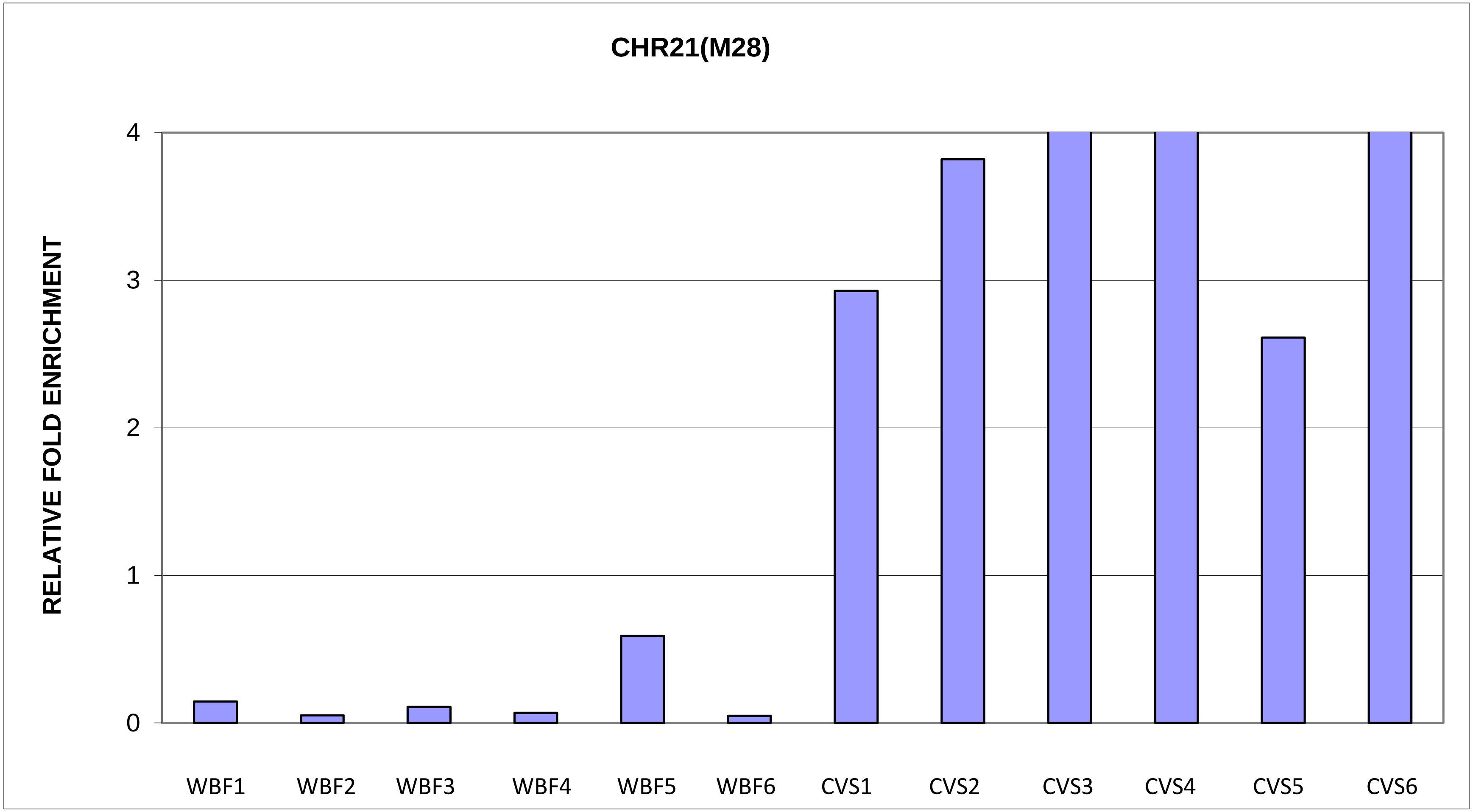

CHR21(M28)
RELATIVE FOLD ENRICHMENT
4
3
2
1
0
WBF1
WBF2
WBF3
WBF4
WBF5
WBF6
CVS1
CVS2
CVS3
CVS4
CVS5
CVS6

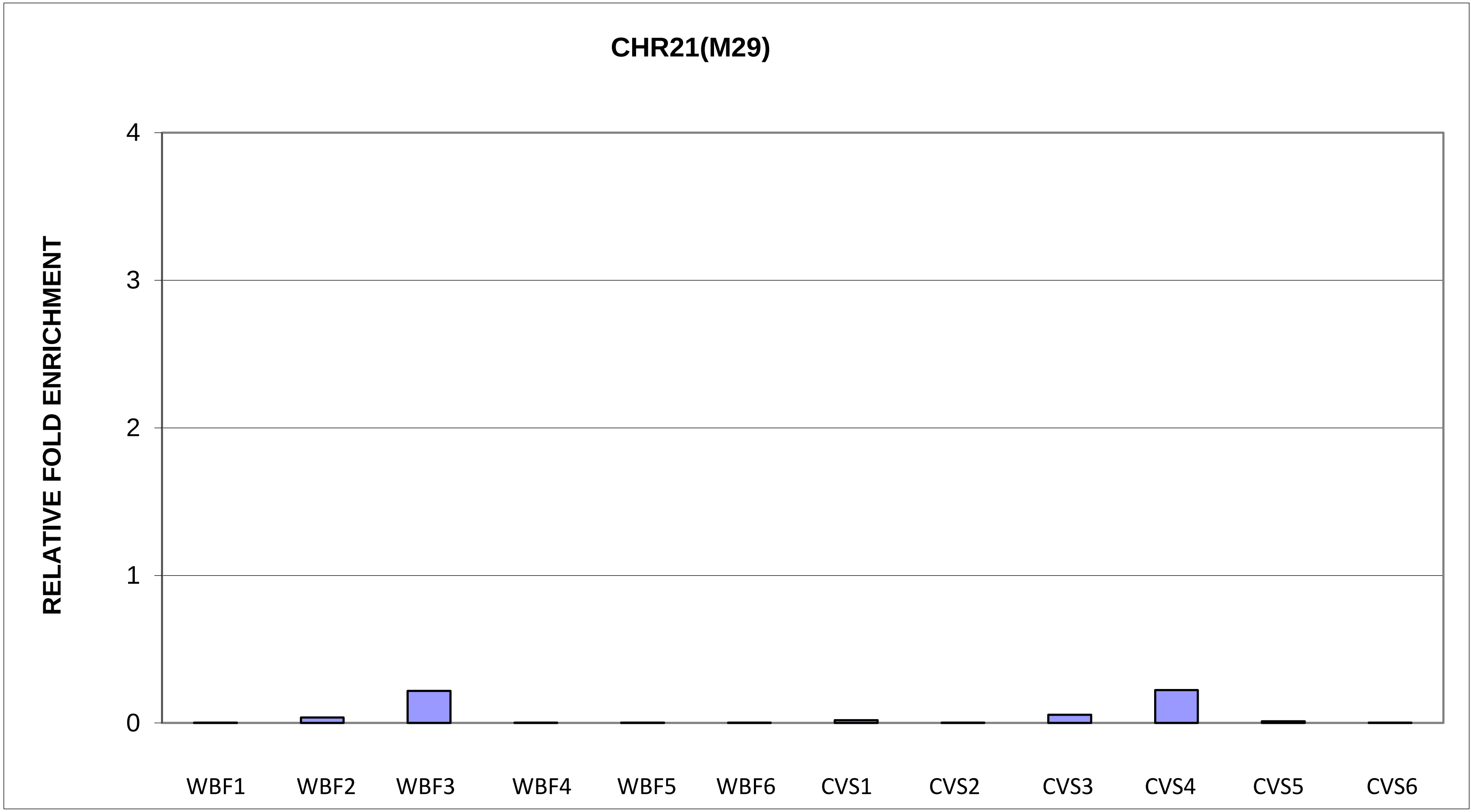
CHR21(M29)
RELATIVE FOLD ENRICHMENT
4
3
2
1
0
WBF1
WBF2
WBF3
WBF4
WBF5
WBF6
CVS1
CVS2
CVS3
CVS4
CVS5
CVS6

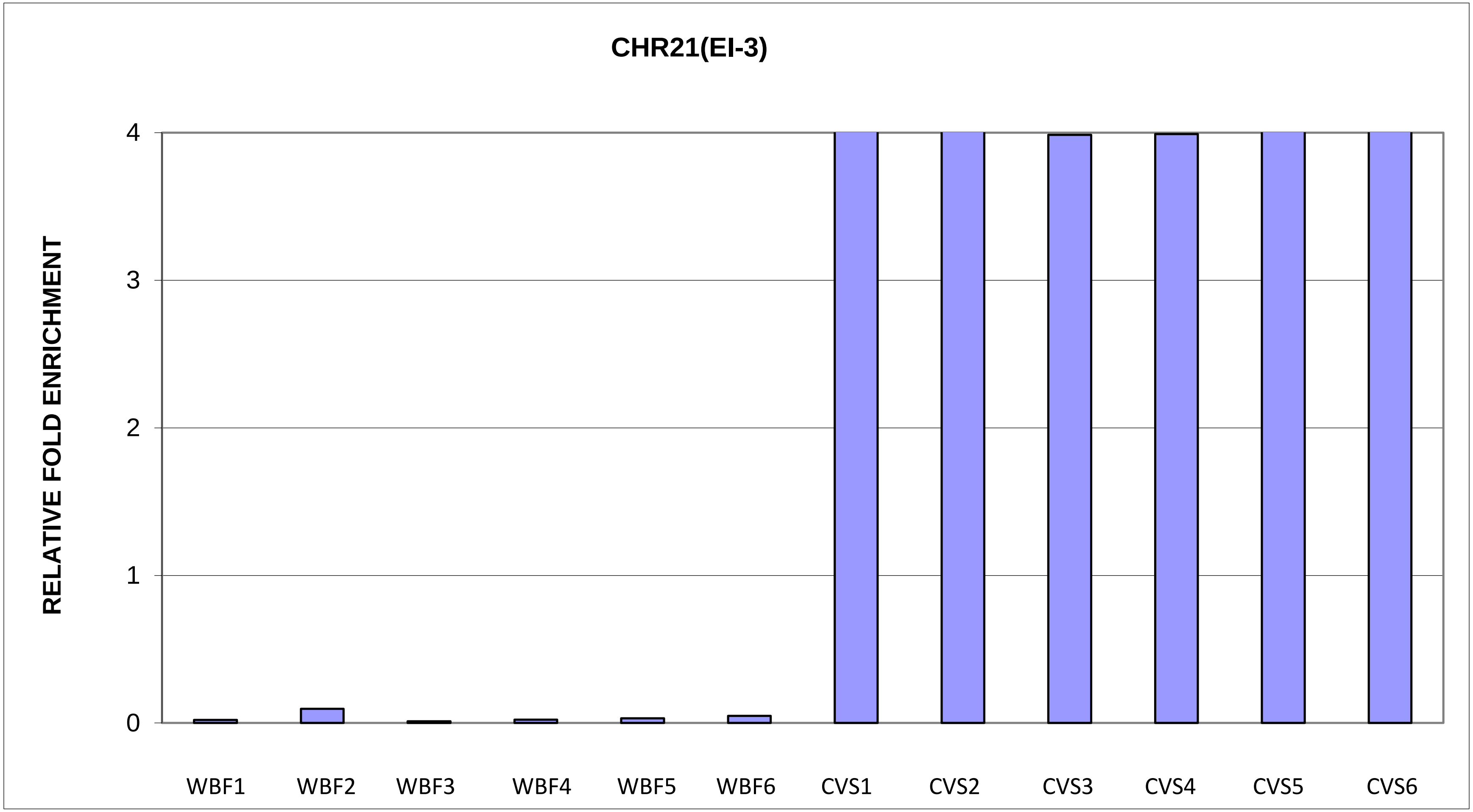

CHR21(EI-3)
RELATIVE FOLD ENRICHMENT
4
3
2
1
0
WBF1
WBF2
WBF3
WBF4
WBF5
WBF6
CVS1
CVS2
CVS3
CVS4
CVS5
CVS6

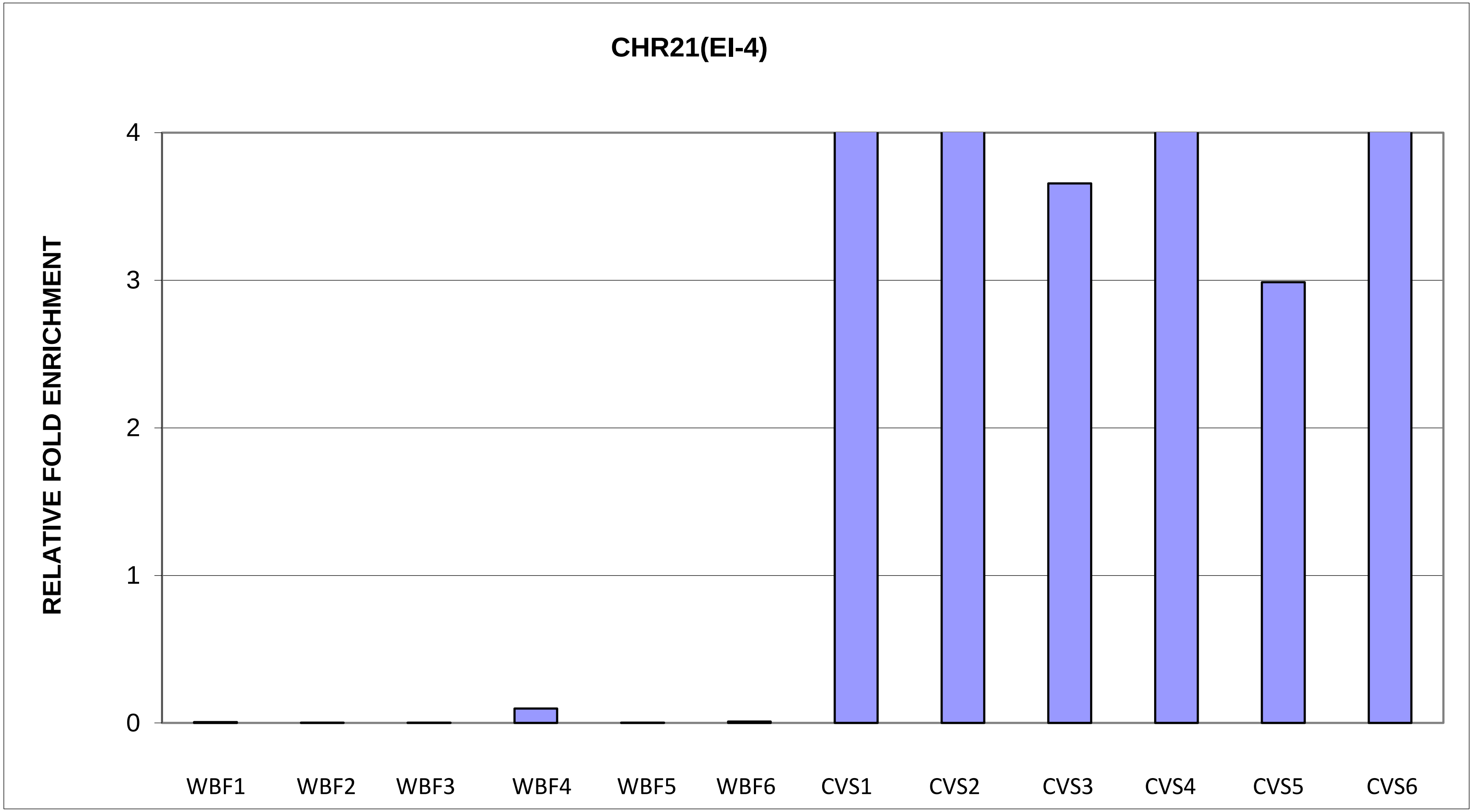
CHR21(EI-4)
RELATIVE FOLD ENRICHMENT
4
3
2
1
0
WBF1
WBF2
WBF3
WBF4
WBF5
WBF6
CVS1
CVS2
CVS3
CVS4
CVS5
CVS6
